# Elucidation of the co-metabolism of glycerol and glucose in *Escherichia coli* by genetic engineering, transcription profiling, and ^13^C metabolic flux analysis

**DOI:** 10.1186/s13068-016-0591-1

**Published:** 2016-08-22

**Authors:** Ruilian Yao, Dewang Xiong, Hongbo Hu, Masataka Wakayama, Wenjuan Yu, Xuehong Zhang, Kazuyuki Shimizu

**Affiliations:** 1 State Key Laboratory of Microbial Metabolism and School of Life Sciences and Biotechnology, Shanghai Jiao Tong University, 800 Dongchuan Road, Shanghai, 200240 China; 2 Institute for Advanced Biosciences, Keio University, 246-2, Mizukami, Kakuganji, Tsuruoka, Yamagata 997-0052 Japan; 3 Instrumental Analysis Center, Shanghai Jiao Tong University, 800 Dongchuan Road, Shanghai, 200240 China

**Keywords:** Glycerol, ^13^C metabolic flux analysis, Carbon catabolite repression, Cofactor, PTS, Transcriptional regulation

## Abstract

**Background:**

Glycerol, a byproduct of biodiesel, has become a readily available and inexpensive carbon source for the production of high-value products. However, the main drawback of glycerol utilization is the low consumption rate and shortage of NADPH formation, which may limit the production of NADPH-requiring products. To overcome these problems, we constructed a carbon catabolite repression-negative ΔptsGglpK* mutant by both blocking a key glucose PTS transporter and enhancing the glycerol conversion. The mutant can recover normal growth by co-utilization of glycerol and glucose after loss of glucose PTS transporter. To reveal the metabolic potential of the ΔptsGglpK* mutant, this study examined the flux distributions and regulation of the co-metabolism of glycerol and glucose in the mutant.

**Results:**

By labeling experiments using [1,3-^13^C]glycerol and [1-^13^C]glucose, ^13^C metabolic flux analysis was employed to decipher the metabolisms of both the wild-type strain and the ΔptsGglpK* mutant in chemostat cultures. When cells were maintained at a low dilution rate (0.1 h^−1^), the two strains showed similar fluxome profiles. When the dilution rate was increased, both strains upgraded their pentose phosphate pathway, glycolysis and anaplerotic reactions, while the ΔptsGglpK* mutant was able to catabolize much more glycerol than glucose (more than tenfold higher). Compared with the wild-type strain, the mutant repressed its flux through the TCA cycle, resulting in higher acetate overflow. The regulation of fluxomes was consistent with transcriptional profiling of several key genes relevant to the TCA cycle and transhydrogenase, namely *gltA*, *icdA*, *sdhA* and *pntA*. In addition, cofactor fluxes and their pool sizes were determined. The ΔptsGglpK* mutant affected the redox NADPH/NADH state and reduced the ATP level. Redox signaling activated the ArcA regulatory system, which was responsible for TCA cycle repression.

**Conclusions:**

This work employs both ^13^C-MFA and transcription/metabolite analysis for quantitative investigation of the co-metabolism of glycerol and glucose in the ΔptsGglpK* mutant. The ArcA regulatory system dominates the control of flux redistribution. The ΔptsGglpK* mutant can be used as a platform for microbial cell factories for the production of biofuels and biochemicals, since most of fuel molecule (e.g., alcohols) synthesis requires excess reducing equivalents.

**Electronic supplementary material:**

The online version of this article (doi:10.1186/s13068-016-0591-1) contains supplementary material, which is available to authorized users.

## Background

With increasing production of biodiesel, a large amount of glycerol is produced as an inevitable byproduct [1]. Significant glycerol surplus has led to a drastic decrease in glycerol prices over the past few years, which makes it an ideal feedstock for the production of high-value products [1–3]. The main drawback of glycerol utilization in *Escherichia coli* is the relatively low carbon source consumption rate, cell growth, and productivity [4]. The main reason for this is the allosteric inhibition of the rate-limiting GLPK by FBP and EIIA^Glc^ under aerobic conditions [5–8]. FBP and EIIA^Glc^ both act to reduce *v*_*max*_ of GLPK, which displays a dimer–tetramer equilibrium in solution [6]. FBP acts both to promote dimer–tetramer assembly and to inactivate the tetramers [7]. The crystal structure of the EIIA^Glc^:GLPK complexes has been determined [8]. GLPK with bound glycerol and ADP forms tetramers in which each GLPK subunit interacts with one EIIA^Glc^ molecule, and the association of the two proteins forms a novel intermolecular binding site for Zn (II) [9]. Genetic modification of the *glpK* gene has resulted in the change in this enzyme that is insensitive to FBP and EIIA^Glc^, allowing improved the glycerol consumption rate [10, 11]. Another obstacle to glycerol utilization is the shortage of NADPH formation because minimal glycolytic flux is reverted from GAP upwards to the oxidative pentose phosphate (PP) pathway. Because NADPH is an important cofactor needed for the production of useful metabolites, co-fermentation of glycerol with glucose has been proposed as an efficient process [4, 12, 13], especially for promoting 1,3-propanediol fermentation for the industrial scale production by DuPont [14]. However, glucose utilization prevents the metabolism of glycerol because of carbon catabolite repression (CCR) [15].

The central players in CCR in *E. coli* are the transcriptional activator Crp, cAMP receptor protein, the signal metabolite cAMP, Cya, and the phosphorylation system (PTS); these systems are involved in transport and/or phosphotransferase reactions of carbohydrates [15]. The PTS in *E. coli* consists of two common cytoplasmic proteins, EI, encoded by *ptsI*, and HPr, encoded by *ptsH*, as well as carbohydrate-specific EII complexes encoded by *crr* and *ptsG* [16]. One metabolic engineering strategy to relax CCR is the inactivation of PTS genes [13, 17, 18]. The part PEP not consumed in glucose transport was canalized to shikimate pathway [17], which is a very important route for the synthesis of aromatic amino acids and natural products [19]. Therefore, we constructed a CCR-negative ΔptsGglpK* mutant by both blocking a key glucose transporter gene *ptsG* and replacing the native *glpK* with *glpK22* from *E. coli* Lin 43 to enhance the glycerol conversion [12]. This mutant can co-consume glycerol and glucose with a faster glycerol assimilation rate than glucose assimilation rate [12]. To reveal the metabolic potential of the ΔptsGglpK* mutant, it is necessary to understand its flux distributions and regulation of the co-metabolism of glycerol and glucose in the mutant.

The fluxome is the final functional output of cell metabolism, and is controlled by genetic regulation, enzyme capability, substrate availability, natural allostery, and metabolic network structures [20, 21]. ^13^C metabolic flux analysis (^13^C-MFA) can accurately determine the fluxes in a metabolic network, which is the most informative tool in quantifying the cellular carbon and energy metabolism and is useful in identifying rate-controlling enzymes [21–23]. ATP and its products ADP/AMP are key regulatory molecules that control enzyme activities in central metabolisms. The limitation of ATP prevents the host from achieving high carbon yields and metabolite production rates. ^13^C-MFA can profile carbon fluxes through most energy generation/consumption pathways, which is the only tool to quantify this problem [24]. Various studies have been reported elucidating ^13^C-MFA using single-labeled substrate, while a limited number of investigations have been reported performing labeling experiments using multiple isotopic tracers [25, 26]. For instance, the tracer mixture, [1,2-^13^C]glucose and [U-^13^C]glutamine led to flux estimates of superior quality in both glycolysis and the TCA cycle in mammalian cells [25].

This study investigated the co-metabolism of glycerol and glucose in *E. coli*, and elucidated the metabolic potential of the ΔptsGglpK* mutant as a chassis for biosynthesis from cheap feedstock. ^13^C-MFA provided rigorous comparison of cell fluxomes between the wild-type and the mutant strain using well-controlled chemostat cultures: (1) various growth rates could be realized by changing the dilution rate; and (2) cell metabolism could be maintained in a metabolic steady-state during labeling experiment [27, 28]. To complement the fluxome studies, we also examined the transcriptional levels of key genes and intracellular pyridine nucleotide pools.

## Results

### Fermentation characteristics of the wild type and the ΔptsGglpK* mutant

**Fig. 1:**
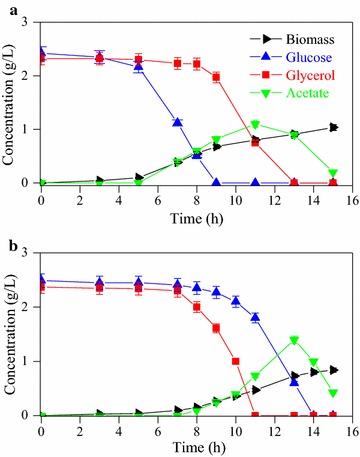
Batch fermentation characteristics of *E. coli* BW25113 (**a**) and the ΔptsGglpK* mutant (**b**). Data represent the mean ± SD from three independent cultures

Figure 1 shows the batch fermentation characteristics of the wild-type *E. coli* (BW25113) and the ΔptsGglpK* mutant. The wild-type strain consumed glucose and glycerol sequentially owing to CCR (Fig. 1a), while the ΔptsGglpK* mutant co-metabolized both glucose and glycerol with faster glycerol consumption than glucose consumption (Fig. 1b; Additional file 1a). By modulating GLPK and the deletion of *ptsG*, the specific glycerol consumption rate in the ΔptsGglpK* mutant was 2.7-fold higher than that in the wild-type strain. To understand the co-metabolism of glycerol and glucose in the ΔptsGglpK* mutant, a detailed analysis was performed in continuous cultures.

Table 1 summarizes the fermentation characteristics of the two strains in the continuous cultures at dilution rates of 0.1 and 0.35 h^−1^. The two strains showed similar fermentation characteristics at the dilution rate of 0.1 h^−1^ in terms of the specific glucose consumption rate, the specific glycerol consumption rate, and biomass concentration without acetate formation. The fermentation characteristics were quite different at the dilution rate of 0.35 h^−1^. In the wild type, more glucose was catabolized than glycerol. The specific glucose consumption rate was 7.2 times higher than that at the lower dilution rate, while the specific glycerol consumption was decreased slightly. In the ΔptsGglpK* mutant, more glycerol was catabolized than glucose (more than tenfold higher). The specific glycerol consumption rate was more than sevenfold higher at the higher dilution rate compared with that at the lower dilution rate, while the specific glucose consumption rate was similar. The biomass concentration was reduced and acetate overflow was significant in both strains at the higher dilution rate. At the dilution rate of 0.35 h^−1^, the specific glucose consumption rate was decreased by 84.7 %, the specific glycerol consumption rate was increased 8.0-fold, and the specific acetate formation rate was increased 1.3-fold in the ΔptsGglpK* mutant compared with the wild type.

**Table 1:** Continuous fermentation characteristics of *E. coli* BW25113 and the ∆ptsGglpK* mutant

Strain	Dilution rate (h^−1^)	Specific glucose consumption rate (mmol/g/h)	Specific glycerol consumption rate (mmol/g/h)	Specific acetate formation rate (mmol/g/h)	Biomass (g/L)
Wild type	0.1	1.03 ± 0.05	2.01 ± 0.10	0	1.08 ± 0.05
∆ptsGglpK*	0.1	0.96 ± 0.04	1.87 ± 0.09	0	1.15 ± 0.05
Wild type	0.35	7.43 ± 0.37	1.72 ± 0.08	6.97 ± 0.35	0.52 ± 0.02
∆ptsGglpK*	0.35	1.14 ± 0.05	13.90 ± 0.62	9.13 ± 0.43	0.46 ± 0.01

Data represent the mean ± SD from three independent cultures

### Metabolic flux analysis

**Fig. 2:**
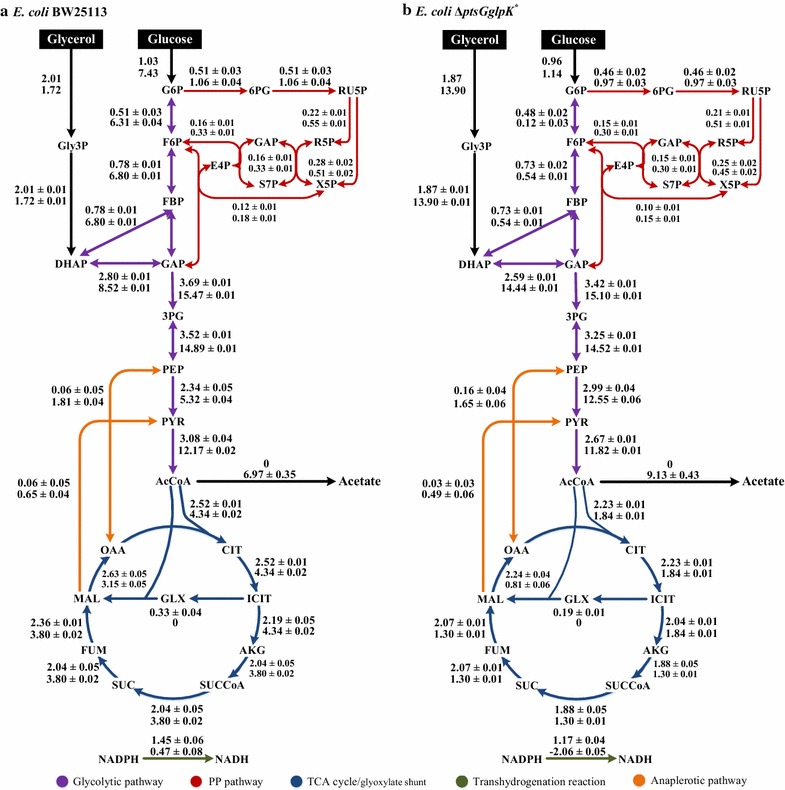
Metabolic flux distributions of *E. coli* BW25113 (**a**) and the ΔptsGglpK* mutant (**b**). Fluxes shown are absolute values (mmol/g/h) (flux ± SD). *Numbers* represent the fluxes at dilution rates of 0.1 (*top*) and 0.35 h^−1^ (*bottom*)

Figure 2 shows the metabolic flux distributions in chemostat cultures of the wild-type strain and the ΔptsGglpK* mutant grown on [1,3-^13^C]glycerol and [1-^13^C]glucose. In the present study, [1-^13^C]glucose was used to identify the flux ratio between the PP pathway and glycolysis at the G6P node, while [1,3-^13^C]glycerol was used to discriminate the labeling patterns originating from labeled glucose. The measured mass isotopomer distribution (MDV) of proteinogenic amino acids and the simulated MDV coincided well (Additional file 2), indicating a good fit and high flux precision. The exchange coefficients are listed in Additional file 3. The flux patterns resemble those of glucose-feeding for the upper part of glycolysis and the PP pathway, and those of both glucose and glycerol co-feeding for the other parts of central metabolism.

First, we compared flux distributions for the two strains at the dilution rate of 0.1 h^−1^. The fluxes were the similar, consistent with the fermentation characteristics. Second, we compared the effects of the dilution rate on the flux distributions of the two strains. In the wild type, as the dilution rate was increased, the absolute fluxes through the PP pathway, glycolytic pathway, anaplerotic pathway and the TCA cycle significantly increased, while the flux through the glyoxylate shunt became negligible. However, the relative distribution of the flux through the TCA cycle decreased from 83 to 47 % (normalized by the substrate consumption rate), while the flux into acetate formation increased. In the ΔptsGglpK* mutant, the absolute fluxes through the PP pathway, glycolytic pathway, and anaplerotic pathway were also enhanced, while the absolute flux through the TCA cycle was 17 % lower than that at the dilution rate of 0.1 h^−1^. The relative flux distribution through the TCA cycle significantly decreased, from 79 to 12 %, while the flux into acetate formation increased. Last, we compared the flux distributions for the two strains at the dilution rate of 0.35 h^−1^. Striking differences were seen between the two strains: in the ΔptsGglpK* mutant as compared with the wild-type strain: (1) the flux through phosphoglucose isomerase decreased by 98.1 %; (2) the flux through GLPDH increased 8.1-fold; (3) the flux through the TCA cycle flux decreased by 57.6 %; and (4) the transhydrogenation flux was reversed, from NADPH → NADH to NADH → NADPH.

### Intracellular pyridine nucleotide and ATP pools

The cofactors such as NADH, FADH_2_ and NADPH play important roles in regulating redox relevant reactions. NADH/FADH_2_ are involved in ATP generation of oxidative phosphorylation [29], while the NADPH is essential for anabolism [30]. Table 2 compares the intracellular pyridine nucleotide and ATP pools of the two strains in two chemostat cultures. At the dilution rate of 0.1 h^−1^, the two strains had the similar intracellular pyridine nucleotide and ATP pools. When the dilution rate was increased, the NADPH and NADP^+^ levels increased (*P* < 0.01 for both) without affecting the NADPH/NADP^+^ ratio in the wild-type strain. In addition, the NADH and NAD^+^ pools and the NADH/NAD^+^ ratio increased (*P* < 0.01 for all) in this strain. In the ΔptsGglpK* mutant, the NADPH level and the NADPH/NADP^+^ ratio decreased (*P* < 0.01 for both), while the concentrations of NADH and NAD^+^ and the NADH/NAD^+^ ratio increased (*P* < 0.01 for all) with the increasing dilution rate. The concentrations of ATP increased at the dilution rate of 0.35 h^−1^ compared with 0.1 h^−1^ in both strains (*P* < 0.01 for both). Compared with the wild-type strain at the dilution rate of 0.35 h^−1^, the NADPH, NADP^+^, NAD^+^ and ATP concentrations and the NADPH/NADP^+^ ratio were lower (*P* < 0.01 for all), and the NADH level and the NADH/NAD^+^ ratio were higher (*P* < 0.1 and *P* < 0.01) in the ΔptsGglpK* mutant at the same dilution rate. The decreased NADPH/NADP^+^ and increased NADH/NAD^+^ ratios indicated the alteration of the cellular redox state in the ΔptsGglpK* mutant.

**Table 2:** Comparison of pyridine nucleotide and ATP pools of *E. coli* BW25113 and the ΔptsGglpK* mutant

Strain	Dilution rate (h^−1^)	NADPH (nmol/mg)	NADP^+^ (nmol/mg)	NADPH/NADP^+^	NADH (nmol/mg)	NAD^+^ (nmol/mg)	NADH/NAD^+^	ATP (nmol/mg)
Wild type	0.1	0.59 ± 0.03	0.45 ± 0.02	1.33 ± 0.09	0.43 ± 0.02	1.33 ± 0.06	0.32 ± 0.02	1193 ± 130
∆ptsGglpK*	0.1	0.59 ± 0.02	0.45 ± 0.01	1.33 ± 0.07	0.43 ± 0.02	1.38 ± 0.07	0.31 ± 0.02	953 ± 131
Wild type	0.35	0.77 ± 0.04	0.67 ± 0.03	1.16 ± 0.08	1.39 ± 0.06	2.76 ± 0.13	0.50 ± 0.03	11,784 ± 583
∆ptsGglpK*	0.35	0.35 ± 0.03	0.43 ± 0.02	0.80 ± 0.06	1.52 ± 0.07	2.17 ± 0.10	0.70 ± 0.05	2378 ± 213

### Relative gene transcription levels

**Fig. 3:**
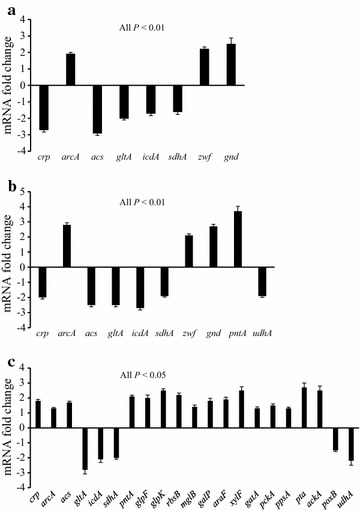
Fold changes of transcription levels of selected genes. **a** *E. coli* BW25113 at the dilution rate of 0.35 h^−1^ compared with 0.1 h^−1^. **b** The ΔptsGglpK* mutant at the dilution rate of 0.35 h^−1^ compared with 0.1 h^−1^. **c** The ΔptsGglpK* mutant compared with the wild type at the dilution rate of 0.35 h^−1^

Figure 3 shows the gene expression data measured by qRT-PCR. In the wild-type strain, the transcription level of the global regulator gene *crp* was downregulated, while *arcA* was upregulated, at the dilution rate of 0.35 h^−1^ as compared with 0.1 h^−1^. Crp induces expression of its target genes, which include transporters and other genes in the TCA cycle, gluconeogenesis and acetate utilization pathway, upon allosteric activation through high cAMP concentrations [31, 32]. ArcA represses many genes involved in the TCA cycle and electron transport [33]. In accordance with the repression of *crp*, the transcription level of the acetate utilization pathway gene *acs* was downregulated (Additional file 4). In accordance with the repression of *crp* and the activation of *arcA*, the transcription levels of *gltA*, *icdA* and *sdhA* in the TCA cycle were downregulated (Additional file 4). The transcription levels of *zwf* and *gnd* in the oxidative PP pathway were upregulated. In the ΔptsGglpK* mutant, the changing patterns of these genes were similar to what was observed for the wild-type strain with the increasing dilution rate (Fig. 3b). In addition, *pntA* showed a higher expression level while *udhA* showed a lower expression level compared with 0.1 h^−1^, implying that excess NADH may be converted to NADPH through the transhydrogenase reaction. Figure 3c compares gene expression levels in the ΔptsGglpK* mutant with the wild-type strain at the dilution rate of 0.35 h^−1^. The transcription levels of global regulator genes *crp* and *arcA* were upregulated. In accordance with the increase in *crp* expression, the transcription levels of transporter genes *glpFK* (glycerol), *rbsB* (ribose), *mglB/galP* (galactose), *araF* (arabinose), *xylF* (xylose), and *gatA* (galactitol), gluconeogenic genes *pckA* and *ppsA*, and acetate utilization pathway gene *acs* were upregulated (Additional file 4). In the *E. coli* PTS^−^ strains, glucose was probably transported through GalP and MglBAC systems [34]. The upregulation of *glpFK* correlates to the increased glycerol consumption rate in the mutant. The overexpression of *glpFK* was also observed in the strain JM101 grown on glycerol as compared to glucose [35]. Compared with the wild type at the dilution rate of 0.35 h^−1^, the transcription levels of *gltA*, *icdA*, *sdhA* and *udhA* were downregulated, while the transcription level of *pntA* was upregulated in the mutant at the same dilution rate. The repression of the TCA cycle was higher in the ΔptsGglpK* mutant than the wild-type strain at the dilution rate of 0.35 h^−1^, indicating that the repression by ArcA was more than the activation by Crp. Compared with the wild type at the dilution rate of 0.35 h^−1^, the transcription levels of *pta* and *ackA* were upregulated, while *poxB* was downregulated in the ΔptsGglpK* mutant at the same dilution rate. This indicates that acetate is mainly synthesized by phosphotransacetylase/acetate kinase pathway in the mutant.

## Discussion

### Central metabolism and CCR

In general, the Δ*pts* mutant has much slower growth without acetate overflow due to the limitation of glucose uptake [16, 32, 34, 36]. By overexpression of glycerol conversion pathway, the ΔptsGglpK* mutant can recover similar growth rate in bioreactors to the wild type if both glycerol and glucose were supplied. At the dilution rate of 0.35 h^−1^, the acetate formation rate in the ΔptsGglpK* mutant becomes even higher than the wild-type strain, indicating the re-organization of carbon and energy (ATP) metabolism for fast biomass production when glucose and glycerol are sufficient. To further elucidate this observation, ^13^C-MFA provides direct information on intracellular flux rerouting and offers great insight into the co-mechanism of glycerol and glucose usage. At the dilution rate of 0.1 h^−1^, the wild type and ΔptsGglpK* mutant strains showed similar fermentation characteristics, intracellular pyridine nucleotide pools, and flux distributions, and CCR was rarely observed without acetate overflow. Since low dilution rate mimics the late stationary phase because of carbon source limitation, this drives *E. coli* cells to reduce its glycolytic flux and scavenge any possible carbon sources including acetate [37]. Similar to *E. coli* growing at the low dilution rate, other slow growing bacteria with weak glycolytic fluxes, such as *Corynebacterium* and *Rhodococcus* [38, 39], also showed no CCR without acetate overflow.

As the dilution rate was increased, the improved carbon source uptake changed the overall flux magnitude, resulting in increased absolute flux values except for the TCA cycle in the ΔptsGglpK* mutant. The fluxes through the PP pathway, glycolytic pathway, and anaplerotic pathway significantly increased to fulfill the demand for biomass synthesis. High carbon source consumption evokes CCR, and a higher fraction of the consumed carbon source was secreted as overflow metabolites [16, 40]. In both strains, the decrease in *crp* expression demonstrated that CCR was activated. As a consequence, cAMP-Crp dependent regulation of *acs* and *gltA* was downregulated. This result is consistent with a previous study on *E. coli* using advanced continuous cultivation methods [32]. The downregulation of *gltA* was in line with the relative flux results, which caused acetate to be accumulated (Additional file 5). Because the main carbon source consumed in the wild-type strain is glucose, the metabolic characteristics are similar to those when using glucose as the sole carbon source. In the Δ*ptsG* mutant, EIIA^Glc^-P accumulates and activates Cya. Then the level of cAMP is enhanced, which increases the level of the cAMP-Crp and activates the transcription of *crp* [41]. Finally, CCR is relaxed and co-consumption of glucose–pentose can be attained [42]. The glycerol metabolism partially induces catabolite repression [43], and the *glpK* mutant strains enhances the power of glycerol to exert this repression [40, 44]. Glycerol or Gly3P cause strong repression of *malT*, although phosphorylation of EIIA^Glc^ is only slightly lowered [43]. The proposed mechanism is that Gly3P inhibits EIIA^Glc^-P-mediated stimulation of Cya, thus lowering the cAMP concentrations [43]. Because the inhibition of GLPK by FBP and EIIA^Glc^ was relieved in the *glpK* mutant [10], fast glycerol consumption increased the transfer of phosphoryl groups from EIIA^Glc^ to produce Gly3P. The resulting decrease in EIIA^Glc^-P in turn reduced Cya activity and cAMP production [40]. Together, deletion of *ptsG* and replacement of *glpK* caused transcriptional repression to a lesser extent than the wild-type strain, as shown by the upregulations of *crp*, transporter genes, gluconeogenic genes and *acs* in the ΔptsGglpK* mutant compared with the wild type growing at the dilution rate of 0.35 h^−1^ (Fig. 2c). This mutant can co-utilize mixtures of glycerol–xylose and glycerol–galactose, but it cannot co-utilize the mixture of glycerol-acetate (Additional file 1).

### Cofactors NADH and FADH_2_ metabolism

**Fig. 4:**
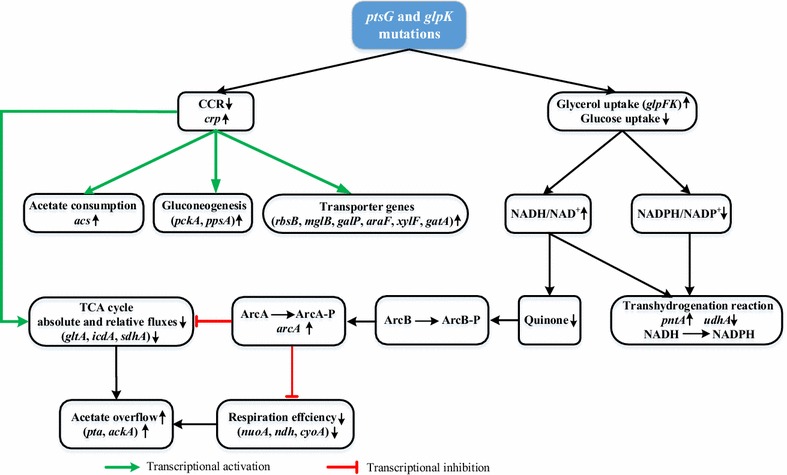
Proposed mechanism of the metabolic effects of *ptsG* and *glpK* mutations in *E. coli*

The cofactors balance can be estimated from the metabolic fluxes [45, 46]. Table 3 summarizes the specific rates of the formation and consumption of cofactors derived from the flux data given in Fig. 2. The major pathway enzymes for supplying NADH are GAPDH and PDH in glycolysis, AKGDH and MDH in the TCA cycle, and transhydrogenase (UdhA) [29]. SDH and GLPDH are the main enzymes for the genesis of FADH_2_ [5, 47]. At the dilution rate of 0.1 h^−1^, the fluxes from the glycolysis accounted for 51 % of the NADH formation, while those from the TCA cycle accounted for 35 % of the NADH formation in the two strains. When the dilution rate was increased, the glycolytic flux increased, producing a large amount of NADH from the glycolysis in both strains. In the case of the ΔptsGglpK* mutant, FADH_2_ formation at GLPDH became significant as the glycerol consumption rate increased at the dilution rate of 0.35 h^−1^ (Table 3). Increased NADH and FADH_2_, as major donors to the electron transport chain, create a highly oxidative environment which is detrimental to aerobically growing *E. coli* cells [48]. NADH allosterically inhibits CS and ICDH [49]. In addition, redox state as a signal activates the ArcA regulatory system. Because the NADH level and the NADH/NAD^+^ ratio were the highest we observed in the ΔptsGglpK* mutant growing at the dilution rate of 0.35 h^−1^, the inhibition of the TCA cycle was more severe than in the wild type, as demonstrated by the upregulation of *arcA* and the downregulation of *gltA*, *icdA* and *sdhA* (Fig. 3c). This suggests that ArcA, more than Crp, regulated the expression levels of the TCA cycle genes when glycerol was being consumed as the main carbon source at the higher dilution rate (Fig. 4). In addition, ArcA exerts a negative control on the TCA cycle fluxes in the aerobic batch culture of *E. coli*, where knockout of ArcA-dependent regulation increased the TCA cycle fluxes by over 60 % [50]. The higher dilution rate mimics the cell growth phase in batch culture. Therefore, the relative flux through CS in the TCA cycle decreased in both strains with increasing dilution rate. Furthermore, the absolute flux through the TCA cycle increased in the wild type, but decreased in the ΔptsGglpK* mutant, indicating strong repression of the TCA cycle when glycerol was being consumed as the main carbon source at the higher dilution rate. Reduction of the TCA cycle flux limited NADH production: the fluxes from the TCA cycle accounted for 19 and 7 % of the NADH formation in the wild type and ΔptsGglpK* strains at the dilution rate of 0.35 h^−1^, respectively. Consequently, this reduced the oxidative stress caused by the oxidation of NADH and FADH_2_, as also implied by Mailloux et al. [48]. Meanwhile, the activated transhydrogenase PntAB converted excess NADH to NADPH and reduced redox imbalance.

**Table 3:** Estimated production and consumption of cofactors and ATP by *E. coli* BW25113 and the ΔptsGglpK* mutant

Strain	Wild type		∆ptsGglpK*	
a NADH				
Dilution rate (h^−1^)	0.1	0.35	0.1	0.35
GLPDH	0	0	0	0
Glycolysis	6.77	27.64	6.09	26.90
TCA cycle	4.67	6.95	4.12	1.30
PP pathway	0	0	0	0
Anaplerotic pathway	0.03	0.33	0.02	0.25
Acetate formation	0	0	0	0
Biomass formation	0.33	1.17	0.33	1.17
Transhydrogenation	1.45	0.47	1.17	−2.26
Oxidative phosphorylation	−13.25	−36.57	−11.72	−28.18
b FADH_2_				
Dilution rate (h^−1^)	0.1	0.35	0.1	0.35
GLPDH	2.01	1.72	1.87	13.90
Glycolysis	0	0	0	0
TCA cycle	2.36	3.80	2.07	1.30
PP pathway	0	0	0	0
Anaplerotic pathway	0	0	0	0
Acetate formation	0	0	0	0
Biomass formation	0	0	0	0
Transhydrogenation	0	0	0	0
Oxidative phosphorylation	−4.37	−5.52	−3.94	−15.20
c NADPH				
Dilution rate (h^−1^)	0.1	0.35	0.1	0.35
GLPDH	0	0	0	0
Glycolysis	0	0	0	0
TCA cycle	2.19	4.34	2.04	1.84
PP pathway	1.02	2.12	0.92	1.94
Anaplerotic pathway	0.03	0.33	0.02	0.25
Acetate formation	0	0	0	0
Biomass formation	−1.80	−6.30	−1.80	−6.30
Transhydrogenation	−1.45	−0.47	−1.17	2.26
Oxidative phosphorylation	0	0	0	0
d ATP				
Dilution rate (h^−1^)	0.1	0.35	0.1	0.35
Glucose uptake	0	0	−0.96	−1.14
Glycerol uptake	−2.01	−1.72	−1.87	−13.90
GLPDH	0	0	0	0
Glycolysis	5.25	13.99	5.68	27.11
TCA cycle	2.04	3.80	1.88	1.30
PP pathway	0	0	0	0
Anaplerotic pathway	0	0	0	0
Acetate formation	0	6.97	0	9.13
Biomass formation	−2.30	−8.05	−2.30	−8.05
Transhydrogenation	0	0	0	0
Oxidative phosphorylation^a^	48.49		43.08	
Oxidative phosphorylation^b^		99.48		
Oxidative phosphorylation^c^				71.57
Maintenance cost	51.47	114.47	45.51	86.37

a NADH. b FADH_2_. c NADPH. d ATP

All the fluxes shown are absolute values (mmol/g/h)

^a^The *P/O* ratios for NADH and FADH_2_ were 3 and 2, respectively

^b^The *P/O* ratios for NADH and FADH_2_ were 2.5 and 1.5, respectively

^c^The *P/O* ratios for NADH and FADH_2_ were 2 and 1, respectively

### Cofactor NADPH metabolism

The major NADPH-generating reactions are G6PDH and 6PGDH in the PP pathway, NADP^+^-dependent ICDH in the TCA cycle, and transhydrogenase (PntAB and UdhA) [45]. At the dilution rate of 0.1 h^−1^, the TCA cycle contributed a large fraction of the supply of NADPH in both strains. At the lower dilution rate, the NADPH required for anabolic demand is small, so the transhydrogenase flux converted excess NADPH to NADH, 45 % for the wild type, and 39 % for the ΔptsGglpK* mutant, respectively. This result is consistent with a previous study on *E. coli* in glucose-limited chemostat cultures. When the dilution rate was increased, the NADPH requirement for biosynthesis increased. The activity of both G6PDH and 6PGDH has been reported to be growth rate dependent; here, we consider the activation of the oxidative PP pathway to be due to the NADPH requirement for anabolic demand, as demonstrated by the increased transcriptional levels of *zwf* and *gnd* in both strains. For the wild type growing at the dilution rate of 0.35 h^−1^, catabolic NADPH production via the PP pathway and TCA cycle almost matched the anabolic demands. For the ΔptsGglpK* mutant growing at the dilution rate of 0.35 h^−1^, these two sources of NADPH seemed to be insufficient. Although about 85 % of glucose passed through the oxidative PP pathway, little glycerol was channeled to the upper glycolysis and PP pathway. Minimal flux from GAP upwards to G6P was also observed in *B. subtilis* grown on glycerol [51]. Thus, the flux from the oxidative PP pathway accounted for 31 % of the NADPH supply in the ΔptsGglpK* mutant at the dilution rate of 0.35 h^−1^. Because of NADH and FADH_2_ accumulation, the TCA cycle was significantly repressed and accounted for only 29 % of the NADPH supply in the ΔptsGglpK* mutant at the dilution rate of 0.35 h^−1^. There was about a 36 % gap to be filled to fulfill the demand for NADPH. The transhydrogenation reaction served this purpose, as also noted by Sauer et al. [45]. In line with the flux results, the transcriptional level of *pntA* was upregulated in the ΔptsGglpK* mutant at the dilution rate of 0.35 h^−1^ compared with the case at 0.1 h^−1^, as well as to the wild type at the dilution rate of 0.35 h^−1^. It has also been reported that expression of *pntAB* was induced when there was a demand for NADPH, and *pntAB* was required for optimal growth on carbon sources whose metabolism does not directly generate NADPH [30].

### Energy (ATP) metabolism

Based on fluxes, the specific ATP formation and consumption rates were estimated in the two strains (Table 3d). *E. coli* can produce ATP from either oxidative phosphorylation or substrate-level phosphorylation such as acetate formation [52]. Since the molar ATP yield from respiration is higher than that from substrate-level phosphorylation, the former is much more efficient than the latter [53]. Since the cells exhibited fully respiratory growth under carbon source limited state [45], we assumed the maximum *P/O* ratio to be the moles of ATP formed per oxygen atom: NADH → 3 ATP and FADH_2_ → 2 ATP at the dilution rate of 0.1 h^−1^ [54]. Based on this assumption, the ATP formation fluxes via oxidative phosphorylation were about 9 times of that via substrate-level phosphorylation in both strains. As the dilution rate was increased, the respiration efficiency was lowered in both strains (Additional file 6). Holms [55] also showed that excess glucose (or other highly sufficient carbon sources) inhibited respiration in *E. coli*. Thus, we set the *P/O* ratio to be 2.5 and 1.5 for oxidizing NADH and FADH_2_ at the dilution rate of 0.35 h^−1^. Since the expression levels of respiratory chains genes were more repressed in the ΔptsGglpK* mutant as compared to the wild-type strain (Additional file 6), we chose the *P/O* ratio to be 2 and 1 for oxidizing NADH and FADH_2_, respectively, in the mutant at the dilution rate of 0.35 h^−1^. In any case, the exact *P/O* ratio per se does not affect the main conclusion of the present work. The ATP provided via oxidative phosphorylation in the ΔptsGglpK* mutant was 28 % lower than that in the wild type. In contrast, more ATP was provided by acetate formation in the ΔptsGglpK* mutant, indicating acetate production became an important source of energy generation through substrate-level phosphorylation. The contribution of ATP from substrate-level phosphorylation was increased from 10 to 14 % in the wild type, while it was increased from 10 to 16 % in the ΔptsGglpK* mutant with the increasing dilution rate.

Acetate overflow is interpreted as a manifestation of the imbalance of carbon uptake/availability and those for energy production and biosynthesis [52]. Why was ATP less generated in the ΔptsGglpK* mutant, where glycerol consumption was dominant as compared to the wild-type strain, where the glucose consumption was dominant at the dilution rate of 0.35 h^−1^, resulting in more acetate overflow? Firstly, the lower expression levels of respiratory chains genes caused lower *P/O* in the ΔptsGglpK* mutant. This led to the reduction in ATP supply via oxidative phosphorylation. Secondly, less NADPH, which is an integral part of the oxidative energy-generating machinery of aerobic organisms, was produced in the ΔptsGglpK* mutant. NADPH helps maintain the reductive environment necessary for oxidative phosphorylation. Without continual supply of NADPH, production of ATP by oxidative phosphorylation cannot proceed effectively [56]. Finally, the activity of PntAB is energy dependent [45], indicating that the ΔptsGglpK* mutant requires more energy input at the dilution rate of 0.35 h^−1^. These could all lead to the decrease of ATP production. As a result, acetate overflow mechanism was activated to provide additional energy supply in the ΔptsGglpK* mutant.

### The potential of the ΔptsGglpK* mutant for the production of biofuels and biochemicals

This study has increased our understanding of the physiological implications of the ΔptsGglpK* mutant, where the information obtained by ^13^C-MFA integrated with transcription/metabolite profiling is useful for the effective manipulation and engineering of *E. coli* for the production of biofuels and biochemicals. ^13^C-MFA revealed that glucose shifted flux from the glycolytic pathway to the oxidative PP pathway to increase NADPH availability in the ΔptsGglpK* mutant, demonstrating the benefit of co-utilization of glycerol and glucose for NADPH supply. In our previous work, we engineered an acetol overproducing strain (HJ05) through overexpressing of *yqhD* and silencing of *gapA* from the ΔptsGglpK* mutant [12]. Because the aldehyde oxidoreductase encoded by *yqhD* utilizes NADPH as a cofactor [57], NADPH availability is one important factor for the acetol production. Compared with utilization of glycerol as a sole carbon source in the control strain, co-utilization of glycerol and glucose in HJ05 increased NADPH availability and acetol production (1.82 g/L).

This study found that the ΔptsGglpK* mutant showed the increased NADH level and the NADH/NAD^+^ ratio. The excess reducing equivalents could be consumed by converting glycerol to more reduced products under microaerobic conditions, such as ethanol and 1,2-propanediol (1,2-PDO) [58, 59]. The 1,2-PDO biosynthesis pathways of *E. coli* using glycerol as a carbon source with glucose as a co-substrate are shown in Additional file 7. The production of 1,2-PDO from glycolytic intermediate DHAP has been well established [59, 60]. Coupling parts of these strategies with our previous work on the efficient production of acetol (an intermediate upstream of 1,2-PDO synthesis) could be an effective approach to increase 1,2-PDO and lower the NADH level and the NADH/NAD^+^ ratio.

## Conclusions

The present investigation shows the power of ^13^C-MFA using multiple isotopic tracers, applied in this case to the co-metabolism of glucose and glycerol in the *E. coli* ΔptsGglpK* mutant. The ΔptsGglpK* mutant preferentially consumed glycerol by reorganization of its fluxomes: fast glycerol consumption in the ΔptsGglpK* mutant affected the redox NADPH/NADH state and reduced the ATP level, activated the ArcA regulatory system, and repressed the TCA cycle, causing acetate overflow. In addition, glucose shifted flux from the glycolytic pathway to the oxidative PP pathway to increase NADPH availability in the ΔptsGglpK* mutant, demonstrating that additional carbon source is usually required to help provide the cofactor and the production of other products from glycerol which is limited. The ΔptsGglpK* mutant can be used as a platform for the production of high-value products, especially those requiring excess reducing equivalents (such as alcohol biofuels).

## Methods

### Strains, culture medium and growth conditions

The strains used in this study are listed in Table 4. The parental strain *E. coli* BW25113 (CGSC 7636) and *E. coli* Lin 43 (CGSC 5511) containing the *glpK22* allele were obtained from the *E. coli* Genetic Stock Center at the Department of Biology, Yale University. The sequence of the *glpK22* gene was found in GenBank under accession no. U41468 [61]. The *ptsG* gene in *E. coli* BW25113 (UniProt: P69786) was disrupted by P1 phage transduction [62].

**Table 4:** Strains used in this study

Strains	Relevant genotype or description	Source or reference
BW25113	*F* ^−^ *λ* ^−^ *rph* ^−*1*^ Δ*araBAD*_*AH33*_ *lacI* ^*q*^ Δ*lacZ*_*WJ16*_ *rrnB*_*T14*_ Δ*rhaBAD*_*LD78*_ *hsdR514*	*E. coli* Genetic Stock Center from Yale University
Lin 43	Hfr(PO2A) *fhuA22*, *ΔphoA8*, *fadL701*(T2R), *relA1*, *glpR2*(*glp* ^*c*^), *pitA10*, *spoT1*, *glpK22*(fbR), *rrnB*-*2*, *mcrB1*, *creC510*	*E. coli* Genetic Stock Center from Yale University
BW25113 Δ*ptsG*	BW25113, *ptsG* ^−^	This study
BW25113 ΔptsGglpK*	BW25113 ∆*ptsG*, *glpK* gene replaced by *glpK22* from strain Lin43	This study

The strains were first pre-cultured in LB medium. The subsequent pre-culture, the batch culture, the main culture and the continuous culture were carried out using M9 minimal medium containing of 2 g/L of glucose and 2 g/L of glycerol. In batch cultures in Additional file 1^1^, the M9 medium contained mixtures of 5 g/L of glycerol and 5 g/L of glucose, 2 g/L of glycerol and 2 g/L of xylose, 2 g/L of glycerol and 2 g/L of galactose, and 2 g/L of glycerol and 0.5 g/L of acetate. The M9 minimal medium contained per liter: 6.81 g Na_2_HPO_4_, 2.99 g KH_2_PO_4_, 0.58 g NaCl and 5.94 g (NH_4_)_2_SO_4_. The following components were filter sterilized and then added (per liter) with 1 ml of 1 M MgSO_4_·7H_2_O, 1 ml of 0.1 mM CaCl_2_·2H_2_O, 1 ml of 1 mg/L thiamine HCl and 10 ml of trace element solution containing (per liter): 0.55 g CaCl_2_·2H_2_O, 1.67 g FeCl_3_·6H_2_O, 0.1 g MnCl_2_·4H_2_O, 0.17 g ZnCl_2_·2H_2_O, 0.043 g CuCl_2_·2H_2_O, 0.06 g CoCl_2_·2H_2_O, and 0.06 g Na_2_M_O_O_4_·2H_2_O. The first pre-culture inoculated from the glycerol stock was grown for 8 h of 10 mL LB medium. The subsequent pre-cultivation was performed by transferring 1 mL of culture broth to a 500-mL baffled Erlenmeyer flasks containing 100 mL of M9 medium. After 12 h, cells were harvested, washed and used to inoculate the main culture. The main culture and continuous culture were conducted in a 2-L fermentor with a working volume of 1 L at 37 °C, 500 rpm with an aeration rate of 1.0 vvm. The pH was controlled at 7.0. The dilution rates in the continuous culture were 0.1 and 0.35 h^−1^. Samples for cell growth, extracellular and intracellular metabolites and real-time quantitative PCR were taken at the steady-state condition (after five residence times).

### Analytical methods

Bacterial growth was monitored by measuring the optical density of the culture broth at 600 nm. Concentrations of glucose, glycerol, xylose, galactose and acetate were measured by high performance liquid chromatography (model 1260, Agilent, Santa Clara, USA) using a cation-exchange column (HPX-87H, Bio-Rad, Hercules, CA) and a differential refractive index (RI) detector. A mobile phase of 5 mM H_2_SO_4_ at 0.5 mL/min flow rate was used and the column was operated at 60 °C.

### Quantification of intracellular cofactors and ATP analysis

Intracellular NADP^+^/NADPH, NAD^+^/NADH were determined by using EnzyChrom NADP^+^/NAD Assay^+^/NADPH and NAD kit (BioAssay Systems, Hayward, CA), following the manufacturer’s instructions. Intracellular ATP was measured by capillary electrophoresis time-of-flight mass spectrometry (CE-TOFMS): Agilent CE capillary electrophoresis system (Agilent Technologies, Germany) and an Agilent G3250AA LC/MSD TOF system (Agilent Technologies, Palo Alto, CA). The measurement conditions have been described elsewhere [63]. The raw CE-TOFMS data were analyzed using MasterHands software version 2.9 [64], and the in vivo metabolite concentrations were quantitated. Data represent the average and standard deviation, which was calculated as average of three technical and two biological replicates. Multiple comparisons among a set of experiments were made by one-way analysis of variance (ANOVA) with the level of significance set at *P* < 0.05.

### Quantitative real-time PCR (qRT-PCR) analysis

Total RNA was isolated using an RNA Extraction Kit (ABigen Corporation, China). Contaminating DNA was removed with RNase-free DNase I (ABigen Corporation, China). The first-strand cDNA was synthesized using PrimeScript™ II 1st Strand cDNA Synthesis Kit (Takara Co. Ltd., China). QRT-PCR was performed with the SYBR^®^ Premix Ex Taq™ Kit (Takara Co. Ltd., China) on an ABI Stepone Real-Time PCR System (Applied Biosystems, USA). The primers used are listed in Additional file 8, and the housekeeping gene 16S rRNA was used to normalize the gene expression data. The PCR conditions were: 95 °C for 4 min, followed by 35 cycles of denaturation at 95 °C for 15 s, annealing at 57 °C for 15 s, and extension at 72 °C for 20 s. Three biological samples were analyzed, and each sample was analyzed three times. The data were averaged and presented as the mean ± standard deviation. Significant differences were determined by one-way analysis of variance (ANOVA). Statistical significance was defined as *P* < 0.05.

## ^13^C-MFA

The labeling experiments were started after taking samples for cell growth, extracellular and intracellular metabolites and real-time quantitative PCR. The unlabeled feeding medium was replaced by an identical medium, a mixture of 50 % [1,3-^13^C]glycerol and 50 % [1-^13^C]glucose. GC–MS analyses of proteinogenic amino acids were made as described previously [65]. In brief, cells were harvested and hydrolyzed in 6 M HCl at 105 °C. The hydrolysate was dried then derivatized using *N*-(*tert*-butyldimethylsilyl)-*N*-methyl-trifluoroacetamide (MTBSTFA) (Sigma-Aldrich, USA). GC–MS analysis was carried out using GC–MS (Hewlett Packard 7890A and 5975C, Agilent Technologies, Santa Clara, CA) equipped with a DB-5MS column (30 m × 0.25 mm × 0.25 μm).

^13^C-MFA was based on the isotopomer mapping matrices (IMMs), and details concerning the general framework of ^13^C-MFA may be found elsewhere [39]. This most frequently employed isotope tracer method detects in 10–15 protein-bound amino acids. The *E. coli* network model used for flux calculation included glucose metabolism, glycerol metabolism, glycolysis, PP pathway, TCA cycle, glyoxylate shunt, lumped biomass formation and transhydrogenation reaction (Additional file 9). The 116 and 120 mass isotopomers were used for the dilution rates at 0.1 and 0.35 h^−1^, respectively. Three external fluxes (glucose, glycerol, acetate) were fitted to the model to estimate 17 or 18 free fluxes for the cases of the dilution rates of 0.35 and 0.1 h^−1^, respectively.

For statistical analysis, 500 simulated measurement data sets of mass distribution were generated by addition of normally distributed measurement noise to the simulated measurement data set corresponding to the best fit flux distribution. Then, from the probability distribution of these re-calculated flux distributions, 90 % confidence intervals could be obtained [65].

## Abbreviations

### Metabolites

AcCoA: acetyl-CoA; AKG: α-ketoglutarate; CIT: citrate; DHAP: dihydroxyacetone phosphate; E4P: erythrose 4-phosphate; F6P: fructose 6-phosphate; FBP: fructose 1,6-bisphosphate; FUM: fumarate; G6P: glucose-6-phosphate; GAP: glyceraldehyde 3-phosphate; GLX: glyoxylate; Gly3P: glycerol-3-phosphate; ICIT: isocitrate; MAL: malate; OAA: oxaloacetate; 3PG: 3-phosphoglyceric acid; 6PG: 6-phosphogluconolactone; PEP: phosphoenolpyruvate; PYR: pyruvate; R5P: ribose 5-phosphate; RU5P: ribulose 5-phosphate; S7P: sedoheptulose 7-phosphate; SUCCoA: succinyl-CoA; SUC: succinate; X5P: xylulose 5-phosphate.

### Proteins (enzymes)

AKGDH: α-ketoglutarate dehydrogenase; CS: citrate synthase; Cya: adenylate cyclase; EI: Enzyme I; EII: Enzyme II; G6PDH: glucose-6-phosphate dehydrogenase; GAPDH: glyceraldehyde 3-phosphate dehydrogenase; GLPDH: glycerol-3-phosphate dehydrogenase; GLPK: glycerol kinase; HPr: Histidine-containing protein; ICDH: isocitrate dehydrogenase; MDH: malate dehydrogenase; 6PGDH: 6-phosphogluconolactone dehydrogenase; PDH: pyruvate dehydrogenase; SDH: succinate dehydrogenase.

### Genes

*ackA*: acetate kinase gene; *acs*: acetyl-CoA synthetase gene; *araF*: arabinose ABC transporter gene; *arcA*: anoxic redox control protein gene; *crp*: cyclic AMP receptor; *galP*: galactose permease gene; *gapA*: glyceraldehyde 3-phosphate dehydrogenase gene; *gatA*: galactitol PTS permease gene; *glpF*: glycerol facilitator gene; *glpK*: glycerol kinase gene; *gltA*: citrate synthase gene; *gnd*: 6-phosphogluconate dehydrogenase gene; *icdA*: isocitrate dehydrogenase gene; *mglB*: galactose ABC transporter gene; *pckA*: phosphoenolpyruvate carboxykinase gene; *pntA*: membrane-bound transhydrogenase gene; *poxB*: pyruvate oxidase gene; *ppsA*: phosphoenolpyruvate synthase gene; *pta*: phosphotransacetylase gene; *ptsG*, *ptsHI*: PTS genes; *rbsB*: ribose ABC transporter gene; *sdhA*: succinate dehydrogenase; *udhA*: soluble transhydrogenase gene; *xylF*: xylose transporter gene; *yqhD*: aldehyde oxidoreductase gene; *zwf*: glucose 6-phosphate dehydrogenase gene.

## Additional files

10.1186/s13068-016-0591-1 Batch fermentation characteristics of the Δ*ptsGglpK** mutant using mixtures of carbon sources. **a** Glycerol-Glucose. **b** Glycerol-Xylose. **c** Glycerol-Galactose. **d** Glycerol-Acetate. Data represent the means ± SD from three independent cultures.
10.1186/s13068-016-0591-1 Experimentally measured and simulated mass distributions (mol %) of amino acid fragment of *E. coli* BW25113 and the Δ*ptsGglpK** mutant at the dilution rates of 0.1 and 0.35 h^−1^.
10.1186/s13068-016-0591-1 The exchange coefficients in *E. coli* BW25113 and the Δ*ptsGglpK** mutant at dilution rates of 0.1 and 0.35 h^−1^.
10.1186/s13068-016-0591-1 Global regulators and their regulated genes.
10.1186/s13068-016-0591-1 The regulation mechanism of the dilution rate on the metabolism of *E. coli* BW25113 (**a**) and the Δ*ptsGglpK** mutant (**b**).
10.1186/s13068-016-0591-1 Fold changes of transcription levels of selected genes. **a** *E. coli* BW25113 at the dilution rate of 0.35 h^−1^ compared with 0.1 h^−1^. **b** The Δ*ptsGglpK** mutant at the dilution rate of 0.35 h^−1^ compared with 0.1 h^−1^. **c** The Δ*ptsGglpK** mutant compared with the wild-type at the dilution rate of 0.35 h^−1^. Asterisks indicate the statistical significance level: *P* < 0.05 (*). *cyoA* cytochrome bo terminal oxidase subunit II gene; *cydA* cytochrome bd-I terminal oxidase subunit I gene; *gapC* split glyceraldehyde 3-phosphate dehydrogenase C gene; *ndh* NADH dehydrogenase NDH-2 gene; *nuoA* NADH dehydrogenase NDH-1 gene; *pgi* phosphoglucose isomerase gene; *rpiB* ribose-5-phosphate isomerase B gene; *talA* transaldolase A gene.
10.1186/s13068-016-0591-1 Metabolic pathways involved in glycerol and glucose dissimilations and biosynthesis of 1,2-propanediol in *E. coli*. Broken lines illustrate multiple steps. *aceE, aceF* pyruvate dehydrogenase genes; *adhE* aldehyde-alcohol dehydrogenase gene; *aldA* lactaldehyde dehydrogenase gene; *dhaKLM* dihydroxyacetone kinase genes; *fbaA, fbaB* fructose bisphosphate aldolase genes; *fucO* 1,2-propanediol reductase gene; *gldA* glycerol dehydrogenase gene; *glk* glucokinase gene; *gloA* glyoxylase type I gene; *gloB* glyoxylase type II gene; *glpD* glycerol-3-phosphate dehydrogenase gene; *ldhA* lactate dehydrogenase gene; *lpdA* lipoamide dehydrogenase gene; *mgsA* methylglyoxal synthase gene; *pflB* pyruvate formate-lyase gene; *pykA, pykF pyruvate kinase gens*; *tpiA* triosephosphate isomerase gene.
10.1186/s13068-016-0591-1 Primers used in this study.
10.1186/s13068-016-0591-1 Metabolic network model of *E. coli* used for ^13^C-MFA.
