# Supplementary material for: Elucidation of the co-metabolism of glycerol and glucose in Escherichia coli by genetic engineering, transcription profiling, and 13C metabolic flux analysis
Source: Biotechnol Biofuels. 2016 Aug 22;9(1):175. doi: 10.1186/s13068-016-0591-1 (PMC4994220; doi:10.1186/s13068-016-0591-1)

Additional file 1. Batch fermentation characteristics of the Δ*ptsGglpK** mutant using mixtures of carbon sources. **a** Glycerol-Glucose. **b** Glycerol-Xylose. **c** Glycerol-Galactose. **d** Glycerol-Acetate. Data represent the means ± SD from three independent cultures.

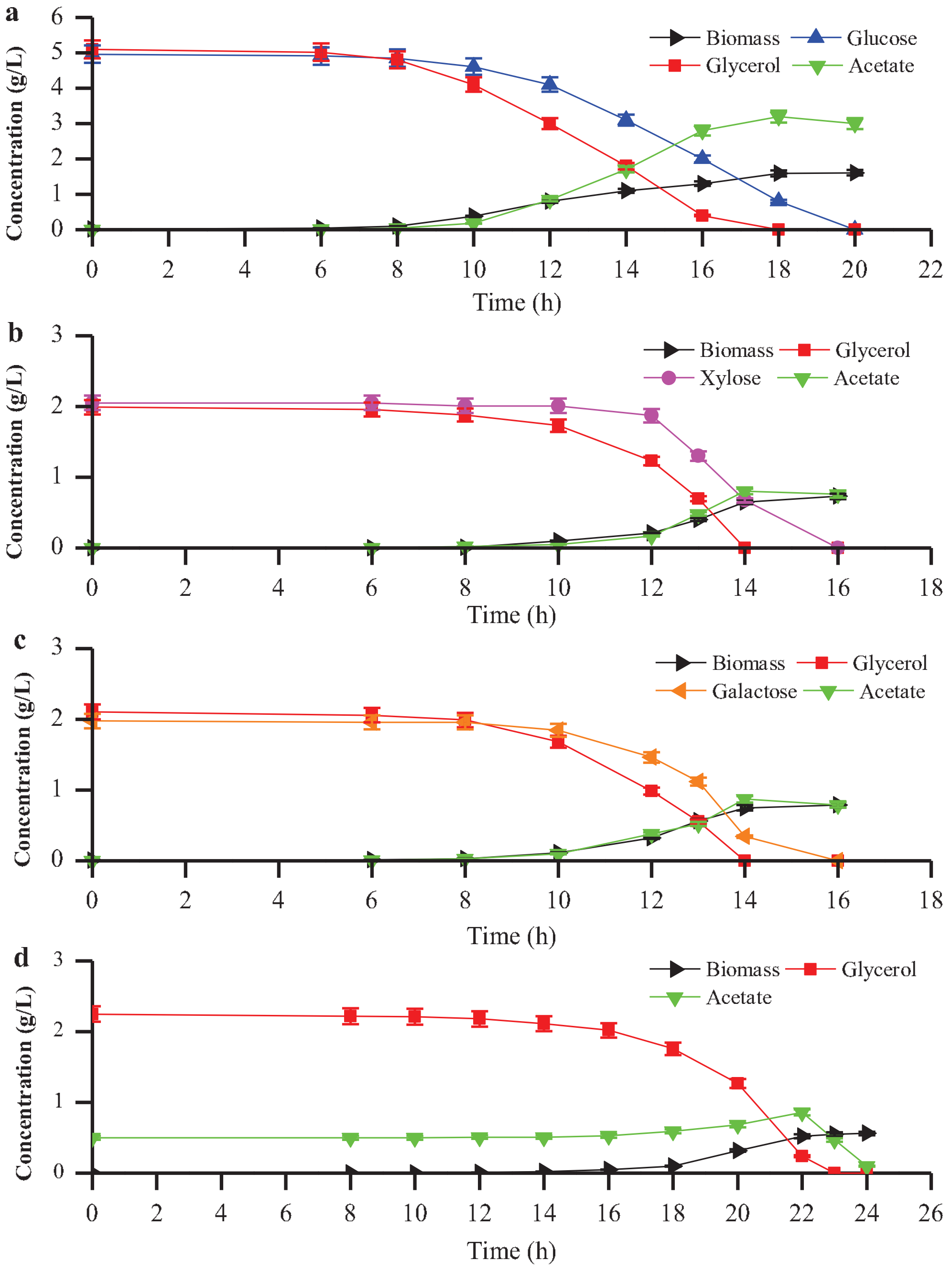

Supplement: Supplementary file 1 — 10.1186/s13068-016-0591-1 Batch fermentation characteristics of the ΔptsGglpK* mutant using mixtures of carbon sources. a Glycerol-Glucose. b Glycerol-Xylose. c Glycerol-Galactose. d Glycerol-Acetate. Data represent the means ± SD from three independent cultures. [file 13068_2016_591_MOESM1_ESM.pdf]
