# Supplementary material for: Elucidation of the co-metabolism of glycerol and glucose in Escherichia coli by genetic engineering, transcription profiling, and 13C metabolic flux analysis
Source: Biotechnol Biofuels. 2016 Aug 22;9(1):175. doi: 10.1186/s13068-016-0591-1 (PMC4994220; doi:10.1186/s13068-016-0591-1)
Supplement: Supplementary file 2 — 10.1186/s13068-016-0591-1 Experimentally measured and simulated mass distributions (mol %) of amino acid fragment of E. coli BW25113 and the ΔptsGglpK* mutant at the dilution rates of 0.1 and 0.35 h−1. [file 13068_2016_591_MOESM2_ESM.pdf]

Additional file 2 Experimentally measured and simulated mass distributions (mol%) of amino acid fragment of *E. coli* BW25113 and the Δ*ptsGglpK** mutant at the dilution rates of 0.1 h^{-1} and 0.35 h^{-1}.

(A) *E. coli* BW25113 at the dilution rate of 0.1 h^{-1}

Fragment		M+0	M+1	M+2	M+3	M+4	M+5
Ala-159	Meas[a]	65.9±0.2	27.2±0.1	5.8±0.1	1.0±0.1		
	Sim[b]	65.7	27.3	5.8	1.0		
Ala-57	Meas	57.1±0.2	22.3±0.2	16.9±0.1	3.6±0.1		
	Sim	56.5	22.0	16.7	3.6		
Ala-85	Meas	59.9±0.2	28.8±0.1	9.2±0.1	2.2±0.1		
	Sim	59.8	28.6	9.0	2.2		
Asp-159	Meas	49.6±0.2	30.9±0.2	14.5±0.1	4.0±0.1	0.9±0.1	
	Sim	49.7	30.8	14.4	4.0	1.0	
Asp-57	Meas	41.8±0.2	29.4±0.1	18.9±0.1	7.4±0.1	2.4±0.1	
	Sim	41.4	29.2	18.8	7.4	2.4	
Asp-85	Meas	45.5±0.2	30.8±0.2	16.6±0.1	5.6±0.1	1.6±0.1	
	Sim	45.2	30.9	16.5	5.5	1.6	
Glu-159	Meas	44.0±0.1	32.2±0.1	16.8±0.1	5.3±0.1	1.4±0.1	0.3±0.0
	Sim	44.1	32.1	16.7	5.4	1.4	0.3
Glu-57	Meas	36.6±0.2	30.3±0.2	20.5±0.1	8.9±0.1	3.0±0.1	0.7±0.1
	Sim	36.5	30.3	20.3	8.8	3.0	0.8
Glu-85	Meas	39.9±0.2	31.7±0.2	18.6±0.2	7.1±0.1	2.2±0.1	0.5±0.1
	Sim	40.1	31.7	18.5	6.9	2.1	0.5
Leu-159	Meas	45.8±0.2	30.5±0.1	17.1±0.1	5.2±0.1	1.2±0.1	0.2±0.1
	Sim	45.7	30.6	17.2	5.2	1.1	0.2
Leu-85	Meas	41.6±0.2	30.4±0.2	19.0±0.1	6.7±0.1	1.8±0.1	0.4±0.1
	Sim	41.6	30.5	18.9	6.8	1.8	0.4
Phe-159	Meas	44.0±0.2	26.9±0.1	16.6±0.1	8.5±0.1	3.2±0.1	0.8±0.1
	Sim	44.1	27.0	16.5	8.4	3.1	0.8
Phe-57	Meas	38.7±0.3	23.6±0.2	18.8±0.2	11.2±0.1	5.5±0.1	2.2±0.1
	Sim	38.4	23.5	18.7	11.1	5.3	2.2
Phe-85	Meas	40.2±0.2	27.0±0.2	18.2±0.2	9.6±0.1	3.9±0.1	1.2±0.1
	Sim	40.1	27.1	18.0	9.5	3.9	1.2
Ser-159	Meas	57.1±0.2	30.2±0.1	10.1±0.1	2.6±0.1		
	Sim	57.4	30.1	9.6	2.5		
Ser-85	Meas	52.5±0.2	30.9±0.1	12.7±0.1	3.9±0.1		
	Sim	52.2	30.6	12.4	3.8		
Tyr-159	Meas	38.2±0.3	27.8±0.2	18.5±0.2	10.0±0.1	4.2±0.1	1.3±0.1
	Sim	38.0	27.7	18.5	9.9	4.2	1.3
Tyr-57	Meas	33.3±0.2	24.4±0.2	20.2±0.1	12.7±0.1	6.7±0.1	2.9±0.0
	Sim	33.1	24.2	19.9	12.4	6.5	2.8
Tyr-85	Meas	34.6±0.2	27.5±0.2	19.7±0.1	11.0±0.1	5.2±0.1	1.9±0.1
	Sim	34.6	27.4	19.6	11.0	5.0	1.8
Val-159	Meas	52.4±0.2	31.7±0.2	12.7±0.1	2.6±0.1	0.6±0.1	

	Sim	52.5	31.6	12.7	2.7	0.5	
Val-57	Meas	45.7±0.2	27.0±0.1	17.6±0.1	7.5±0.1	2.2±0.1	
	Sim	45.5	26.8	17.6	7.6	2.1	
Val-85	Meas	47.6±0.2	31.8±0.2	15.2±0.2	4.4±0.1	1.0±0.1	
	Sim	47.8	31.8	15.1	4.2	1.0	

(B) *E. coli* BW25113 at the dilution rate of 0.35 h^{-1}

Fragment		M+0	M+1	M+2	M+3	M+4	M+5
Ala-159	Meas[a]	71.1±0.2	23.4±0.2	4.7±0.1	0.8±0.1		
	Sim[b]	71.1	23.4	4.7	0.8		
Ala-57	Meas	63.4±0.1	24.1±0.1	10.3±0.1	2.2±0.1		
	Sim	63.2	23.9	10.1	2.2		
Ala-85	Meas	64.9±0.2	25.5±0.1	7.8±0.1	1.8±0.1		
	Sim	64.7	25.3	7.9	1.8		
Asp-159	Meas	54.4±0.2	29.8±0.1	12.0±0.1	3.2±0.1	0.6±0.1	
	Sim	54.4	29.7	12.0	3.2	0.7	
Asp-57	Meas	46.5±0.1	30.4±0.2	15.9±0.1	5.5±0.1	1.7±0.1	
	Sim	46.4	30.4	15.8	5.4	1.6	
Asp-85	Meas	49.6±0.2	30.2±0.2	14.5±0.1	4.5±0.1	1.2±0.1	
	Sim	49.5	30.1	14.4	4.6	1.2	
Glu-159	Meas	48.7±0.2	31.6±0.1	14.2±0.1	4.1±0.1	1.0±0.1	0.3±0.1
	Sim	48.8	31.5	14.3	4.2	1.0	0.2
Glu-57	Meas	41.7±0.2	31.4±0.2	17.7±0.2	6.7±0.1	1.9±0.1	0.6±0.1
	Sim	41.7	31.3	17.7	6.6	2.0	0.5
Glu-85	Meas	44.4±0.2	31.5±0.2	16.3±0.1	5.7±0.1	1.7±0.1	0.4±0.1
	Sim	44.4	31.5	16.4	5.7	1.6	0.4
Leu-159	Meas	52.0±0.2	31.2±0.2	13.1±0.2	3.1±0.1	0.4±0.1	0.1±0.1
	Sim	52.1	31.1	13.2	3.1	0.5	0.1
Leu-85	Meas	47.4±0.1	31.2±0.1	15.6±0.1	4.6±0.1	1.1±0.1	0.2±0.1
	Sim	47.4	31.2	15.5	4.6	1.1	0.2
Phe-159	Meas	51.2±0.2	30.4±0.2	13.5±0.1	4.0±0.1	0.8±0.1	0.1±0.1
	Sim	51.2	30.4	13.5	3.9	0.8	0.1
Phe-57	Meas	45.7±0.2	29.7±0.2	16.4±0.1	6.1±0.1	1.7±0.1	0.4±0.1
	Sim	45.6	29.7	16.3	6.1	1.8	0.4
Phe-85	Meas	46.4±0.1	30.7±0.1	15.9±0.1	5.3±0.1	1.4±0.1	0.3±0.1
	Sim	46.6	30.6	15.7	5.3	1.4	0.3
Ser-159	Meas	61.6±0.1	27.5±0.1	8.8±0.1	2.2±0.1		
	Sim	61.4	27.3	8.8	2.1		
Ser-57	Meas	55.3±0.2	27.3±0.2	13.4±0.1	4.0±0.1		
	Sim	54.7	26.9	13.3	3.9		
Ser-85	Meas	56.3±0.2	28.5±0.2	11.8±0.1	3.4±0.1		
	Sim	55.9	28.3	11.6	3.3		
Tyr-159	Meas	44.2±0.2	31.4±0.1	16.5±0.1	5.8±0.1	1.6±0.1	0.5±0.1

	Sim	44.1	31.4	16.5	5.9	1.6	0.4
Tyr-57	Meas	39.4±0.2	30.1±0.2	18.7±0.2	8.1±0.1	3.0±0.1	0.8±0.1
	Sim	39.3	30.2	18.6	8.1	2.8	0.8
Tyr-85	Meas	40.2±0.2	31.3±0.2	18.3±0.1	7.4±0.1	2.4±0.1	0.6±0.1
	Sim	40.1	31.1	18.3	7.4	2.4	0.6
Val-159	Meas	60.0±0.2	29.5±0.1	8.9±0.1	1.4±0.1	0.2±0.1	
	Sim	60.0	29.4	8.8	1.5	0.2	
Val-57	Meas	53.3±0.2	29.1±0.2	13.0±0.1	3.7±0.1	0.9±0.1	
	Sim	53.3	29.1	12.9	3.8	0.9	
Val-85	Meas	54.6±0.2	30.1±0.2	11.8±0.1	2.8±0.1	0.6±0.1	
	Sim	54.6	30.2	11.7	2.9	0.6	

(C) *E. coli* Δ*ptsGglpK*[*] mutant at the dilution rate 0.1 h^{-1}

Fragment		M+0	M+1	M+2	M+3	M+4	M+5
Ala-159	Meas[a]	65.2±0.1	28.1±0.1	5.7±0.1	1.0±0.1		
	Sim[b]	65.2	28.0	5.7	1.0		
Ala-57	Meas	57.6±0.1	21.3±0.1	17.5±0.1	3.6±0.1		
	Sim	56.9	21.0	17.2	3.6		
Ala-85	Meas	59.5±0.2	29.3±0.2	9.1±0.1	2.2±0.1		
	Sim	59.3	29.2	8.9	2.2		
Asp-159	Meas	48.2±0.2	32.0±0.2	14.7±0.1	4.2±0.1	0.9±0.1	
	Sim	48.1	31.9	14.6	4.2	1.0	
Asp-57	Meas	40.6±0.2	30.0±0.2	19.4±0.2	7.5±0.1	2.5±0.1	
	Sim	40.2	29.8	19.2	7.6	2.5	
Asp-85	Meas	43.9±0.2	31.9±0.1	16.7±0.1	5.7±0.1	1.8±0.1	
	Sim	43.8	31.7	16.7	5.7	1.7	
Glu-159	Meas	42.8±0.1	32.0±0.1	17.5±0.1	5.9±0.1	1.6±0.1	0.3±0.1
	Sim	42.8	32.1	17.5	5.7	1.5	0.3
Glu-57	Meas	35.5±0.2	30.4±0.2	20.9±0.2	9.1±0.1	3.2±0.1	0.9±0.1
	Sim	35.5	30.4	20.9	9.1	3.1	0.9
Glu-85	Meas	38.9±0.2	31.7±0.1	19.3±0.1	7.3±0.1	2.3±0.1	0.6±0.1
	Sim	38.9	31.7	19.2	7.3	2.3	0.6
Leu-159	Meas	44.3±0.2	30.5±0.2	18.1±0.2	5.6±0.1	1.2±0.1	0.2±0.1
	Sim	44.6	30.6	18.0	5.6	1.1	0.2
Leu-85	Meas	40.6±0.2	30.3±0.2	19.5±0.1	7.2±0.1	2.0±0.1	0.4±0.1
	Sim	40.5	30.4	19.6	7.1	1.9	0.4
Phe-159	Meas	42.1±0.2	26.7±0.2	17.3±0.2	9.2±0.1	3.8±0.1	0.9±0.1
	Sim	42.3	26.6	17.2	9.1	3.7	0.9
Phe-57	Meas	37.3±0.2	23.3±0.2	18.8±0.1	11.9±0.1	6.0±0.1	2.7±0.1
	Sim	37.1	23.1	18.7	11.8	5.9	2.5
Phe-85	Meas	38.4±0.2	26.7±0.2	18.6±0.1	10.2±0.1	4.6±0.1	1.5±0.1
	Sim	38.4	26.6	18.6	10.2	4.4	1.4
Ser-159	Meas	57.0±0.2	30.7±0.1	9.8±0.1	2.5±0.1		

	Sim	57.1	30.5	9.5	2.5		
Ser-85	Meas	52.3±0.2	31.4±0.2	12.5±0.1	3.9±0.1		
	Sim	51.9	31.0	12.3	3.8		
Tyr-159	Meas	36.4±0.2	27.3±0.2	19.1±0.1	10.6±0.1	5.0±0.1	1.6±0.1
	Sim	36.4	27.2	19.0	10.6	4.7	1.5
Tyr-57	Meas	32.5±0.2	23.9±0.2	19.8±0.1	13.3±0.1	7.1±0.1	3.4±0.1
	Sim	32.0	23.7	19.8	13.0	7.0	3.2
Tyr-85	Meas	33.1±0.1	27.1±0.1	20.2±0.1	11.8±0.1	5.7±0.2	2.1±0.2
	Sim	33.1	26.8	20.1	11.7	5.6	2.0
Val-159	Meas	51.7±0.2	32.2±0.2	13.0±0.1	2.6±0.1	0.5±0.1	
	Sim	51.9	32.1	13.0	2.6	0.4	
Val-57	Meas	45.6±0.2	26.8±0.1	17.6±0.1	8.0±0.1	2.1±0.1	
	Sim	45.4	26.5	17.5	7.9	2.1	
Val-85	Meas	47.2±0.2	32.2±0.1	15.4±0.1	4.3±0.1	1.0±0.1	
	Sim	47.2	32.2	15.3	4.2	1.0	

(D) *E. coli* BW25113 Δ*ptsGglpK** mutant at the dilution rate 0.35 h^{-1}

Fragment		M+0	M+1	M+2	M+3	M+4	M+5
Ala-159	Meas[a]	60.0±0.2	32.9±0.1	5.7±0.1	1.3±0.1		
	Sim[b]	60.2	32.8	5.7	1.2		
Ala-85	Meas	55.1±0.2	33.5±0.1	8.9±0.1	2.5±0.1		
	Sim	54.8	33.3	9.0	2.5		
Asp-159	Meas	42.7±0.2	31.6±0.1	19.3±0.1	5.1±0.1	1.3±0.1	
	Sim	42.4	31.6	19.2	5.2	1.4	
Asp-57	Meas	36.0±0.2	24.6±0.1	21.4±0.1	13.4±0.1	4.6±0.1	
	Sim	35.6	24.0	20.9	13.2	4.5	
Asp-85	Meas	38.6±0.3	31.2±0.2	20.9±0.1	7.0±0.1	2.3±0.1	
	Sim	38.5	31.2	20.7	6.9	2.2	
Glu-159	Meas	39.5±0.2	33.7±0.1	19.5±0.1	5.5±0.1	1.5±0.1	0.3±0.1
	Sim	39.6	33.6	19.6	5.5	1.5	0.3
Glu-57	Meas	30.9±0.2	27.6±0.1	23.8±0.1	12.2±0.2	4.2±0.1	1.4±0.1
	Sim	30.9	27.5	23.7	12.1	4.2	1.3
Glu-85	Meas	36.0±0.2	32.8±0.1	21.1±0.1	7.4±0.1	2.3±0.1	0.5±0.1
	Sim	36.0	32.8	21.0	7.3	2.3	0.5
Leu-159	Meas	39.6±0.3	29.1±9.1	22.0±0.2	7.8±0.1	1.3±0.1	0.2±0.1
	Sim	39.6	29.0	22.0	7.8	1.4	0.2
Leu-85	Meas	36.0±0.2	28.7±0.1	23.0±0.2	9.3±0.2	2.4±0.2	0.5±0.1
	Sim	36.0	28.7	23.0	9.3	2.4	0.6
Phe-159	Meas	42.9±0.2	31.0±0.1	17.1±0.2	6.2±0.2	2.5±0.1	0.4±0.1
	Sim	42.7	31.1	17.1	6.2	2.5	0.4
Phe-57	Meas	37.4±0.1	22.1±0.1	19.2±0.1	13.8±0.1	5.0±0.1	2.5±0.1
	Sim	37.1	22.0	19.1	13.7	5.0	2.5
Phe-85	Meas	39.0±0.2	30.7±0.1	18.7±0.2	7.6±0.1	3.3±0.1	0.7±0.1

	Sim	38.8	30.7	18.7	7.6	3.2	0.8
Ser-159	Meas	52.2±0.2	34.7±0.2	10.2±0.1	2.9±0.1		
	Sim	51.9	34.5	10.2	2.9		
Ser-85	Meas	47.7±0.1	34.9±0.1	13.0±0.1	4.4±0.1		
	Sim	47.2	34.4	13.0	4.3		
Tyr-159	Meas	36.8±0.2	31.2±0.1	19.4±0.1	8.2±0.1	3.6±0.1	0.9±0.1
	Sim	36.8	31.1	19.4	8.2	3.5	0.9
Tyr-57	Meas	32.3±0.2	23.0±0.2	20.2±0.1	14.7±0.1	6.5±0.1	3.3±0.1
	Sim	32.0	22.7	20.0	14.6	6.4	3.2
Tyr-85	Meas	33.7±0.2	30.6±0.1	20.6±0.2	9.6±0.1	4.3±0.2	1.3±0.1
	Sim	33.4	30.4	20.6	9.6	4.3	1.3
Val-159	Meas	46.3±0.2	33.7±0.1	16.7±0.1	2.7±0.1	0.7±0.1	
	Sim	46.1	33.7	16.8	2.8	0.6	
Val-57	Meas	40.3±0.2	23.6±0.2	19.7±0.1	13.3±0.1	3.1±0.1	
	Sim	39.9	23.4	19.5	13.0	3.2	
Val-85	Meas	417±0.2	33.4±0.1	18.8±0.1	4.8±0.1	1.3±0.1	
	Sim	41.9	33.3	18.7	4.7	1.2	

[a]Meas are the values measured experimentally, reported as mean ± SD, which are uncorrected mass isotopomer abundances.

[b]Sim are values simulated by the isotope model.
