# Supplementary material for: Elucidation of the co-metabolism of glycerol and glucose in Escherichia coli by genetic engineering, transcription profiling, and 13C metabolic flux analysis
Source: Biotechnol Biofuels. 2016 Aug 22;9(1):175. doi: 10.1186/s13068-016-0591-1 (PMC4994220; doi:10.1186/s13068-016-0591-1)
Supplement: Supplementary file 3 — 10.1186/s13068-016-0591-1 The exchange coefficients in E. coli BW25113 and the ΔptsGglpK* mutant at dilution rates of 0.1 and 0.35 h−1. [file 13068_2016_591_MOESM3_ESM.pdf]

Additional file 3 The exchange coefficients in *E. coli* BW25113 and the Δ*ptsGglpK** mutant at the dilution rates of 0.1 h^{-1} and 0.35 h^{-1}.

	Wild type		Δ*ptsGglpK**	
	0.1 h^{-1}	0.35 h^{-1}	0.1 h^{-1}	0.35 h^{-1}
G6P ↔ F6P	0.39 ± 0.05	0.88 ± 0.05	0.92 ± 0.05	0.39 ± 0.05
F6P + ATP ↔ FBP	0.54 ± 0.04	0.95 ± 0.04	0.70 ± 0.03	0.05 ± 0.04
FBP ↔ DHAP + GAP	0.86 ± 0.03	0.44 ± 0.03	0.89 ± 0.03	0.38 ± 0.04
3PG ↔ PEP	0.67 ± 0.05	0.55 ± 0.05	0.18 ± 0.07	0.25 ± 0.04
DHAP ↔ GAP	0.38 ± 0.05	0.86 ± 0.05	0.62 ± 0.05	0.00 ± 0.05
Gly3P ↔ DHAP	0.16 ± 0.05	0.13 ± 0.05	0.30 ± 0.06	0.51 ± 0.05
FUM ↔ MAL	0.95 ± 0.02	0.56 ± 0.05	0.42 ± 0.06	0.94 ± 0.00
MAL ↔ OAA	0.88 ± 0.02	0.95 ± 0.04	0.95 ± 0.06	0.95 ± 0.00
Ru5P ↔ X5P	0.47 ± 0.04		0.24 ± 0.05	0.10 ± 0.04
Ru5P ↔ R5P	0.86 ± 0.05		0.48 ± 0.06	0.50 ± 0.04
R5P + X5P ↔ GAP + S7P	0.41 ± 0.05	0.46 ± 0.04	0.30 ± 0.05	0.75 ± 0.05
X5P + E4P ↔ F6P + GAP	0.19 ± 0.05	0.48 ± 0.05	0.25 ± 0.06	0.08 ± 0.05
S7P + GAP ↔ E4P + F6P	0.95 ± 0.04	0.93 ± 0.03	0.64 ± 0.06	0.04 ± 0.04
PEP + CO_2 ↔ OAA	0.71 ± 0.02	0 ± 0.01	0.51 ± 0.03	0.95 ± 0.00
