# Supplementary material for: Elucidation of the co-metabolism of glycerol and glucose in Escherichia coli by genetic engineering, transcription profiling, and 13C metabolic flux analysis
Source: Biotechnol Biofuels. 2016 Aug 22;9(1):175. doi: 10.1186/s13068-016-0591-1 (PMC4994220; doi:10.1186/s13068-016-0591-1)
Supplement: Supplementary file 4 — 10.1186/s13068-016-0591-1 Global regulators and their regulated genes. [file 13068_2016_591_MOESM4_ESM.pdf]

Additional file 4 Global regulators and their regulated genes.

Global Regulators	Regulation	Metabolic Pathway Genes
Crp/Cya	+	*aceEF*, *acnAB*, *acs*, *focA*, *fumA*, *fur*, *gltA*, *malT*, *manXYZ*, *mdh*, *mlc*, *pckA*, *pflB*, *pgk*, *ptsG*, *sdhCDAB*, *sucABCD, fruBKA, manXYZ, mtlA, rbsB, mglB, araF,* *gatA*, *xylB*, *glpFKX*, *galP*
	–	*cyaA*, *lpdA, rpoS*
ArcA/B	+	*cydAB*, *focA*, *pflB*
	–	*aceBAK*, *aceEF*, *acnAB*, *cyoABCDE*, *fumAC, gltA, icdA, lpdA, mdh, nuoABCEFGHIJKLMN*, *pdhR*, *sodA*, *sdhCDAB, sucABCD*
