# Supplementary material for: Elucidation of the co-metabolism of glycerol and glucose in Escherichia coli by genetic engineering, transcription profiling, and 13C metabolic flux analysis
Source: Biotechnol Biofuels. 2016 Aug 22;9(1):175. doi: 10.1186/s13068-016-0591-1 (PMC4994220; doi:10.1186/s13068-016-0591-1)

Additional file 5. The regulation mechanism of the dilution rate on the metabolism of *E. coli* BW25113 (**a**) and the Δ*ptsGglpK** mutant (**b**).

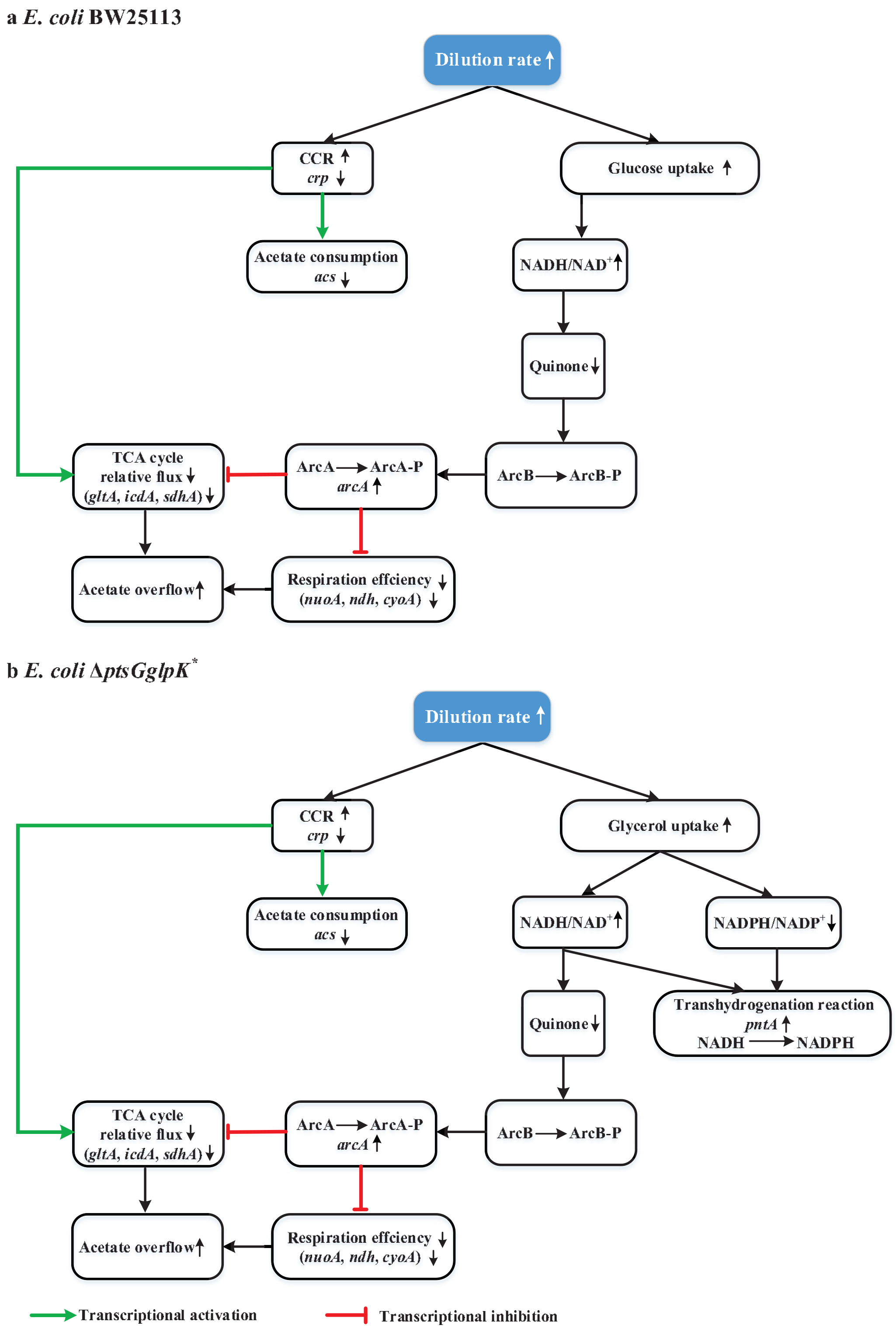

Supplement: Supplementary file 5 — 10.1186/s13068-016-0591-1 The regulation mechanism of the dilution rate on the metabolism of E. coli BW25113 (a) and the ΔptsGglpK* mutant (b). [file 13068_2016_591_MOESM5_ESM.pdf]
