# Supplementary material for: Elucidation of the co-metabolism of glycerol and glucose in Escherichia coli by genetic engineering, transcription profiling, and 13C metabolic flux analysis
Source: Biotechnol Biofuels. 2016 Aug 22;9(1):175. doi: 10.1186/s13068-016-0591-1 (PMC4994220; doi:10.1186/s13068-016-0591-1)

Additional file 6. Fold changes of transcription levels of selected genes. **a** *E. coli* BW25113 at dilution rate of 0.35 h^{-1} compared with 0.1 h^{-1}. **b** The Δ*ptsGglpK*[*] mutant at dilution rate 0.35 h^{-1} compared with 0.1 h^{-1}. **c** The Δ*ptsGglpK*[*] mutant compared with the wild-type at dilution rate 0.35 h^{-1}. Asterisks indicate the statistical significance level: *P*-value < 0.05 (*). *cyoA*: cytochrome bo terminal oxidase subunit II gene; *cydA*: cytochrome bd-I terminal oxidase subunit I gene; *gapC*: split glyceraldehyde 3-phosphate dehydrogenase C gene; *ndh*: NADH dehydrogenase NDH-2 gene; *nuoA*: NADH dehydrogenase NDH-1 gene; *pgi*: phosphoglucose isomerase gene; *rpiB*: ribose-5-phosphate isomerase B gene; *talA*: transaldolase A gene.

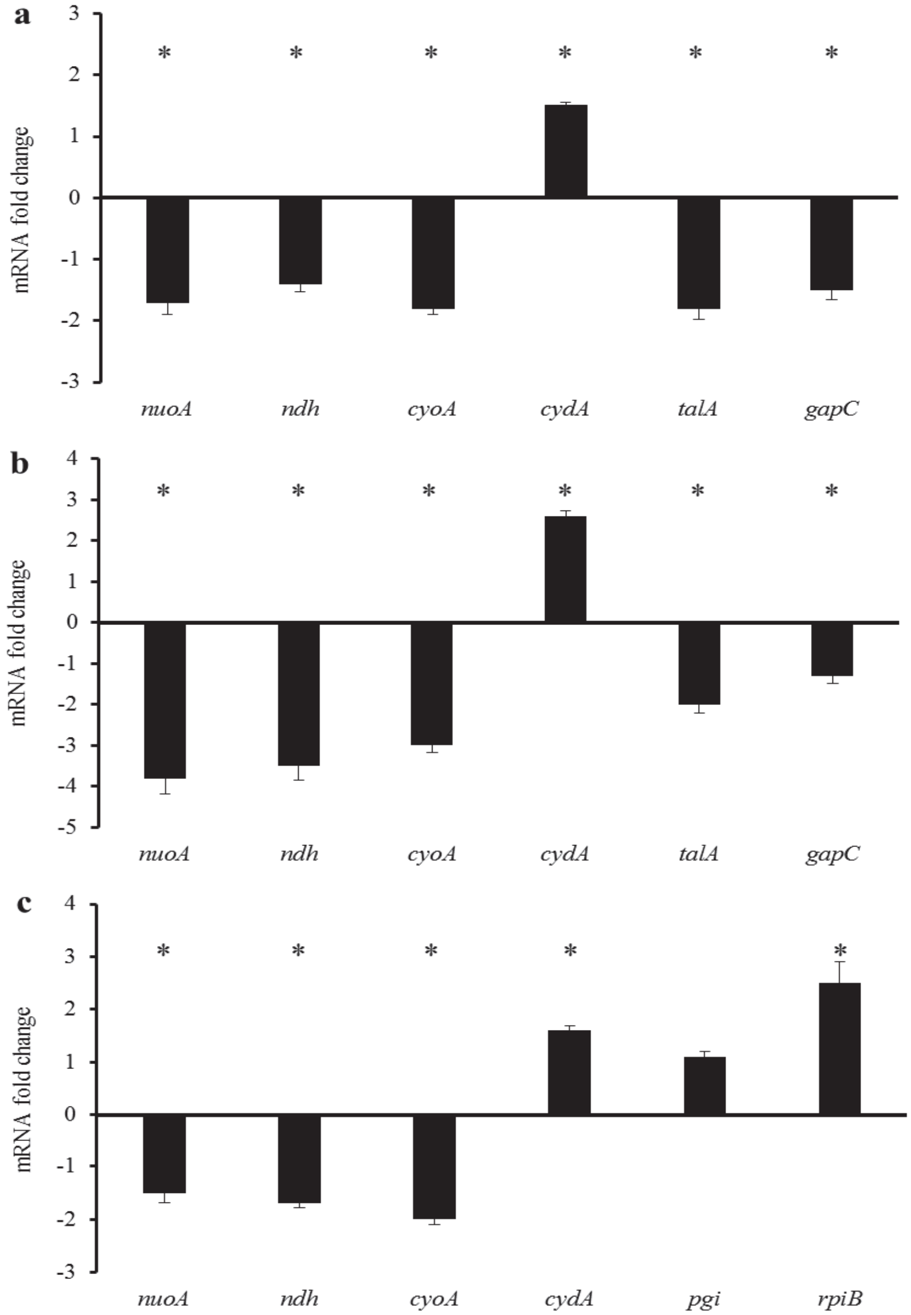

Supplement: Supplementary file 6 — 10.1186/s13068-016-0591-1 Fold changes of transcription levels of selected genes. a E. coli BW25113 at the dilution rate of 0.35 h−1 compared with 0.1 h−1. b The ΔptsGglpK* mutant at the dilution rate of 0.35 h−1 compared with 0.1 h−1. c The ΔptsGglpK* mutant compared with the wild-type at the dilution rate of 0.35 h−1. Asterisks indicate the statistical significance level: P < 0.05 (*). cyoA cytochrome bo terminal oxidase subunit II gene; cydA cytochrome bd-I terminal oxidase subunit I gene; gapC split glyceraldehyde 3-phosphate dehydrogenase C gene; ndh NADH dehydrogenase NDH-2 gene; nuoA NADH dehydrogenase NDH-1 gene; pgi phosphoglucose isomerase gene; rpiB ribose-5-phosphate isomerase B gene; talA transaldolase A gene. [file 13068_2016_591_MOESM6_ESM.pdf]
