# Supplementary material for: Elucidation of the co-metabolism of glycerol and glucose in Escherichia coli by genetic engineering, transcription profiling, and 13C metabolic flux analysis
Source: Biotechnol Biofuels. 2016 Aug 22;9(1):175. doi: 10.1186/s13068-016-0591-1 (PMC4994220; doi:10.1186/s13068-016-0591-1)

Additional file 7. Metabolic pathways involved in glycerol and glucose dissimilations and biosynthesis of 1,2-propanediol in *E. coli*. Broken lines illustrate multiple steps. *aceE, aceF*: pyruvate dehydrogenase genes; *adhE*: aldehyde-alcohol dehydrogenase gene; *aldA*: lactaldehyde dehydrogenase gene; *dhaKLM*: dihydroxyacetone kinase genes; *fbaA, fbaB*: fructose bisphosphate aldolase genes; *fucO*: 1,2-propanediol reductase gene; *gldA*: glycerol dehydrogenase gene; *glk*: glucokinase gene; *gloA*: glyoxylase type I gene; *gloB*: glyoxylase type II gene; *glpD*: glycerol-3-phosphate dehydrogenase gene; *ldhA*: lactate dehydrogenase gene; *lpdA*: lipoamide dehydrogenase gene; *mgsA*: methylglyoxal synthase gene; *pflB*: pyruvate formate-lyase gene; *pykA, pykF: pyruvate kinase gens*; *tpiA*: triosephosphate isomerase gene.

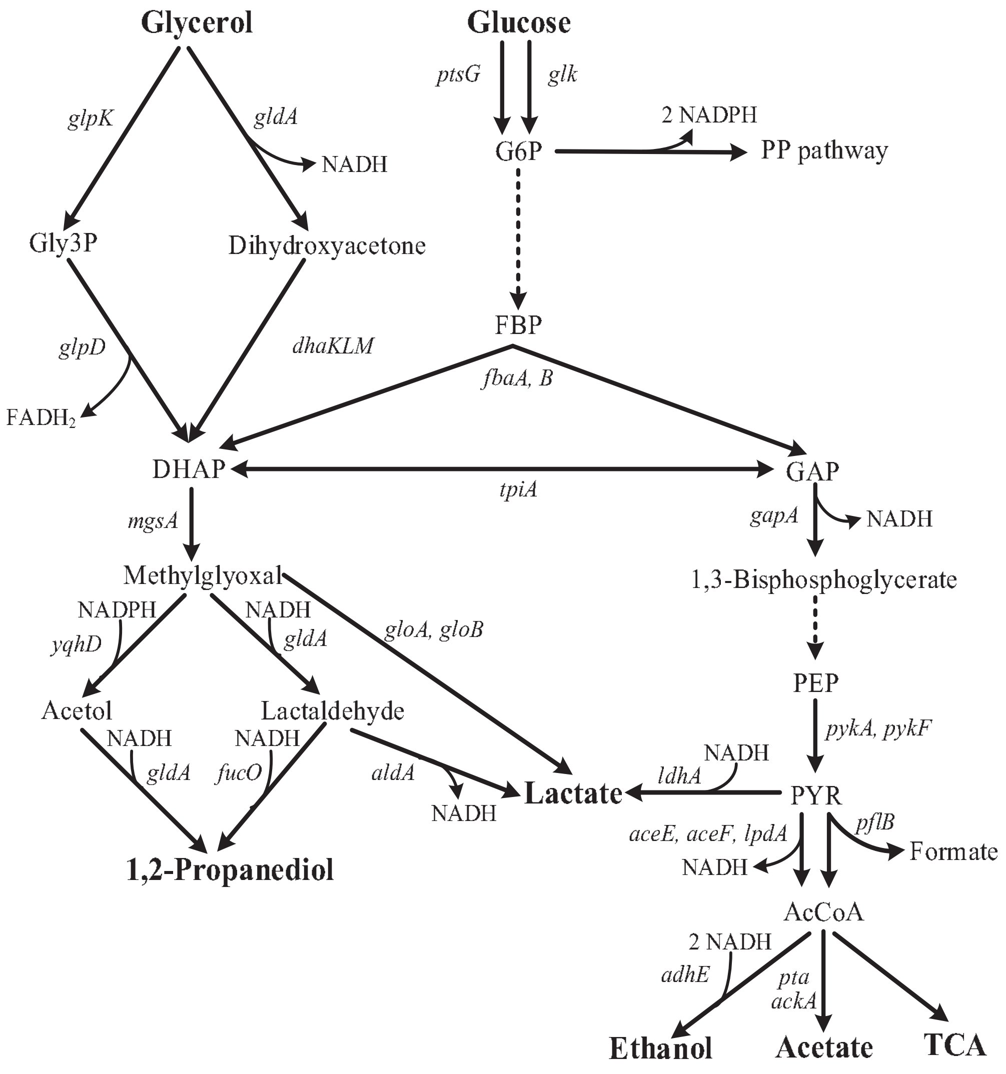

Supplement: Supplementary file 7 — 10.1186/s13068-016-0591-1 Metabolic pathways involved in glycerol and glucose dissimilations and biosynthesis of 1,2-propanediol in E. coli. Broken lines illustrate multiple steps. aceE, aceF pyruvate dehydrogenase genes; adhE aldehyde-alcohol dehydrogenase gene; aldA lactaldehyde dehydrogenase gene; dhaKLM dihydroxyacetone kinase genes; fbaA, fbaB fructose bisphosphate aldolase genes; fucO 1,2-propanediol reductase gene; gldA glycerol dehydrogenase gene; glk glucokinase gene; gloA glyoxylase type I gene; gloB glyoxylase type II gene; glpD glycerol-3-phosphate dehydrogenase gene; ldhA lactate dehydrogenase gene; lpdA lipoamide dehydrogenase gene; mgsA methylglyoxal synthase gene; pflB pyruvate formate-lyase gene; pykA, pykF pyruvate kinase gens; tpiA triosephosphate isomerase gene. [file 13068_2016_591_MOESM7_ESM.pdf]
