# Supplementary material for: Elucidation of the co-metabolism of glycerol and glucose in Escherichia coli by genetic engineering, transcription profiling, and 13C metabolic flux analysis
Source: Biotechnol Biofuels. 2016 Aug 22;9(1):175. doi: 10.1186/s13068-016-0591-1 (PMC4994220; doi:10.1186/s13068-016-0591-1)
Supplement: Supplementary file 8 — 10.1186/s13068-016-0591-1 Primers used in this study. [file 13068_2016_591_MOESM8_ESM.pdf]

Additional file 8 Primers used in this study.

Primer	Sequence
crp-F	CCCATCCAAGAGCACGCTTA
crp-R	CACTTCACAGGCGGTTTTCG
arcA-F	CGCCATGCTTCACTTCTGTG
arcA-R	GCGATGATTTCCGGCGTATC
gltA-F	ACTCTCGGTTCAAAAGGTGTG
gltA-R	GTAGTTAGAATCGGTCGCCAG
icdA-F	ACACTGGCAAAGAGATCGTC
icdA-R	CAGGGCGTCAGAAATGTAGTC
sdhA-F	GTTGAAAGTGATCCGCGAGC
sdhA-R	GAAGTTGGCAGAAACAGCCG
zwf-F	AGTCATGGAGAAACCGCTGG
zwf-R	GGGAGTTAGCAAAACGCAGC
gnd-F	GAAAGATCAGCGTGTTGCCG
gnd-R	CGCCGTAGTTCAGATCCCAG
acs-F	CGGTAAGCCAAAAGGTGTGC
acs-R	GCCTTCAAACATCAGCGTGG
pntA-F	GCGGAAATGGAACTCTTTGC
pntA-R	TTCACGGGTAATTAGCTTCGG
glpK-F	TAACGACGCCTACGATTCCG
glpK-R	CCCACGAGTCAGACCGAAAA
rbsB-F	ATAACGTACTGGGCGGCAAA
rbsB-R	CAAAATCTGCTGGCTGGCTG
mlgB-F	CATGTTATTCGGTGCCGCTG
mglB-R	CTTTCGCCAGCAATACGTCG
araF-F	ATTAAGATTGCCGTGCCGGA
araF-R	TTTAGTCGCCGCCATCATCA
gatA-F	TCGGTAATGAGATGCTCGCC
gatA-R	ATCGCCTCACAATGCGGTAT
pckA-F	GATGTTGCGGTGTTCTTCGG
pckA-R	ACGTTTTCCAGCAACGCATC
ppsA-F	ACGCTGCATTCACAGGGTAA
ppsA-R	TTCGATGCGGTTCATTTCGC
16S-F	TGGTAGTCCACGCCGTAAAC

16S-R	GTTCTTCGCGTTGCATCG
udhA-F	ACAGCGACTCAATTCTCAGC
udhA-R	TCCACTTTTACATCCATACCGC
galP-F	CATGTATTACGCGCCGAAAA
galP-R	TGGCAAGTACGTTGGTCAGG
pta-F	ACAATGTTGATCCGGCGAAG
pta-R	CATATCGATCGCACGAGTCG
ackA-F	CTGGTTCTGAACTGCGGTAGTTC
ackA-R	GGCAGGTGGAAACATTCGG
poxB-F	AAAAGCCGATCGCAAGTTTC
poxB-R	GGTGAATGGCTTTCTCGCTC
talA-F	CTCAAAATCGTACCCGGTCG
talA-R	TACAAGTCCACCAGATGGCG
gapC-F	CAACGACACCATTGTTTCCG
gapC-R	TCATCGTGCCGACTTCTATCC
pgi-F	ACTAACGGTCAGCACGCGTT
pgi-R	TCAGAGAGCGGGTTATGGGT
rpiB-F	GAGAGGTTGATGGCGGGATT
rpiB-R	AGGTTCGCTACAGACGACCG
nuoA-F	TCATCACTGGGCATTCGCTA
nuoA-R	CCATGCGAACAGATACAGCG
ndh-F	AAATACTCCGCCAACCTGGG
ndh-R	CGCCAAGTTTCGTTAGCTCG
cyoA-F	CGACGAGAAGCCCATTACCA
cyoA-R	TACCCAGACGCGGAATGAAG
cydA-F	TGCTATTGAAGCCGAGTGGG
cydA-R	GAGAGTACGCCTTCATCCCG
