# Supplementary material for: Elucidation of the co-metabolism of glycerol and glucose in Escherichia coli by genetic engineering, transcription profiling, and 13C metabolic flux analysis
Source: Biotechnol Biofuels. 2016 Aug 22;9(1):175. doi: 10.1186/s13068-016-0591-1 (PMC4994220; doi:10.1186/s13068-016-0591-1)
Supplement: Supplementary file 9 — 10.1186/s13068-016-0591-1 Metabolic network model of E. coli used for 13C-MFA. [file 13068_2016_591_MOESM9_ESM.pdf]

Additional file 9 Metabolic network model of *E. coli* used for ^{13}C-MFA.

**Glycolysis**	
v1	GLC (abcdef) + ATP → G6P (abcdef) + ADP (Δ*ptsGglpK**)
	GLC (abcdef) + PEP → G6P (abcdef) + PYR (wild type)
v2	G6P (abcdef) ↔ F6P (abcdef)
v30	F6P (abcdef) + ATP ↔ FBP (abcdef)
v3	FBP (abcdef) ↔ DHAP (cba) + GAP (def)
v36	DHAP (abc) ↔ GAP (abc)
v4	GAP (abc) + NAD + PI → 3PG (abc) + NADH + ATP
v5	3PG (abc) ↔ PEP (abc)
v6	PEP (abc) → PYR(abc) + ATP
**Glycerol metabolism**	
v38	Glycerol (abc) + ATP → Gly3P (abc)
v32	Gly3P (abc) + FADH ↔ DHAP (abc) + $FADH_2$
**TCA Cycle**	
v7	PYR (abc) + CoA + NAD → AcCoA (bc) + CO_2 (a) + NADH
v16	AcCoA (AB) + OAA (abcd) → CIT (dcbBAa)
v17	CIT (abcdef) → ICIT (abcdef)
v18	ICIT (abcdef) + NADP → AKG (abcde) + CO_2 (f) + NADPH
v19	AKG (abcde) + NAD + CoA → SUCCoA (bcde) + CO_2 (a)+ NADH
v20	SUCCoA (abcd) → SUCC (abcd) + ATP
v21	SUCC (abcd) + FADH → FUM (½ abcd+½ dcba) + $FADH_2$
v22	FUM (abcd) ↔ MAL (½ abcd+½ dcba)
v23	MAL (abcd) + NAD ↔ OAA (abcd) + NADH
**Pentose Phosphate Pathway**	
v9	G6P (abcdef) + NADP → 6PG (abcdef) + NADPH
v10	6PG (abcdef) + NADP → Ru5P (bcdef) + CO_2 + NADPH
v11	Ru5P (abcde) ↔ X5P (abcde)
v12	Ru5P (abcde) ↔ R5P (abcde)
v13	R5P (ABCDE) + X5P(abcde) ↔ GAP (cde) + S7P (abABCDE)
v14	X5P (abcde) + E4P (ABCD) ↔ F6P (abABCD) + GAP (cde)

v15 S7P (ABCDEFG) + GAP (abc) ↔ E4P (DEFG) + F6P (ABCabc)

Glyoxylate Shunt

v33 ICIT (ABCDEF) + AcCoA (ab) → MAL (ABba) + SUCC (½ fcde+½ edcf)

Amphibolic Reactions

v24 PEP (abc) + CO2 ↔ OAA (abcd)

v25 MAL (abcd) + NAD(P) → PYR (abc) + CO_2 + NAD(P)H

Acetic Acid Formation

v8 AcCoA (ab) → AC (ab) + ATP

Biomass Formation

v27 0.165 G6P + 0.069 F6P + 0.61 R5P + 0.439 E4P + 0.104 GAP + 1.655 m3PG + 0.929 PEP + 3.502 PYR + 2.441 AcCoA + 1.76 OAA + 1.55 AKG + 17.995 NADPH → Biomass + 2.672 CO_2 + 3.349 NADH

Transport

v28 AC → AC.ext

v29 Biomass → Biomass.ext

v31 Glycerol.ext → Glycerol

v26 GLC.ext → GLC

v37 CO_2 → CO_2.ext

Transhydrogantion

v34 NADH + 1/2 O_2 → 3 ATP

v39 $FADH_2$ + 1/2 O_2 → 2 ATP

Transhydrogenation

v35 NAD + NADPH ↔ NADH + NADP

Amino Acid Biosynthesis

B1 PYR (abc) → ALA (abc)

B2	AKG (abcde) → ARG (abcde)
B2B	AKG (abcde) + CO_2 (f) → ARG (abcdef)
B3	OAA (abcd) → ASP (abcd)
B4	AKG (abcde) → GLU (abcde)
B5	3PG (abc) → GLY (ab)
B6	R5P (abcde) → HIS (edcba)
B7	PYR (ABC) + OAA (abcd) → ILE (abBcdC)
B8	2 PYR（ABC+abc） + AcCoA (ef) → LEU (efBbcC)
B9	PYR (ABC) + OAA (abcd) → LYS (½ abcdCB+½ ABCdcb)
B10	OAA (abcd) → MET (abcd)
B11	2 PEP (abc+ABC) + E4P(defg) → PHE (ABCbcedfg)
B12	AKG (abcde) → PRO (abcde)
B13	3PG (abc) → SER (abc)
B14	OAA (abcd) → THR (abcd)
B15	2 PEP (abc+ABC) + E4P(defg) → TYR (ABCbcdefg)
B16	2 PYR (abc+ABC) → VAL (ABbcC)

The free fluxes: v25, v29 and v30 for two strains at the dilution rate of 0.35 h^{-1};

v25, v29, v30 and v33 for two strains at the dilution rate of 0.1 h^{-1}.
